# Transcriptome analysis reveals differences in cell cycle, growth and migration related genes that distinguish fibroblasts derived from pre-invasive and invasive breast cancer

**DOI:** 10.3389/fonc.2023.1130911

**Published:** 2023-04-06

**Authors:** Wei Bin Fang, Marcela Medrano, Paige Cote, Mike Portsche, Vinamratha Rao, Yan Hong, Fariba Behbod, Jennifer R. Knapp, Clark Bloomer, Janelle Noel-Macdonnell, Nikki Cheng

**Affiliations:** ^1^ Department of Pathology and Laboratory Medicine, University of Kansas Medical Center, Kansas City, KS, United States; ^2^ Department of Cancer Biology, University of Kansas Medical Center, Kansas City, KS, United States; ^3^ Center for Genes Environment and Health, National Jewish Health, Denver, CO, United States; ^4^ Kansas Intellectual and Developmental Disabilities Research Center, University of Kansas Medical Center, Kansas City, KS, United States; ^5^ Biostatistics and Epidemiology Core, Health Services and Outcomes Research Children’s Mercy Hospital, Kansas City, MO, United States; ^6^ Department of Pediatrics, University of Missouri-Kansas City (UMKC) School of Medicine, Kansas City, MO, United States

**Keywords:** breast cancer, ductal carcinoma *in situ*, invasive ductal carcinoma, fibroblasts, stroma, transcriptome, cell cycle, invasion

## Abstract

**Background/Introduction:**

As the most common form of pre-invasive breast cancer, ductal carcinoma *in situ* (DCIS) affects over 50,000 women in the US annually. Despite standardized treatment involving lumpectomy and radiation therapy, up to 25% of patients with DCIS experience disease recurrence often with invasive ductal carcinoma (IDC), indicating that a subset of patients may be under-treated. As most DCIS cases will not progress to invasion, many patients may experience over-treatment. By understanding the underlying processes associated with DCIS to IDC progression, we can identify new biomarkers to determine which DCIS cases may become invasive and improve treatment for patients. Accumulation of fibroblasts in IDC is associated with disease progression and reduced survival. While fibroblasts have been detected in DCIS, little is understood about their role in DCIS progression.

**Goals:**

We sought to determine 1) whether DCIS fibroblasts were similar or distinct from normal and IDC fibroblasts at the transcriptome level, and 2) the contributions of DCIS fibroblasts to breast cancer progression.

**Methods:**

Fibroblasts underwent transcriptome profiling and pathway analysis. Significant DCIS fibroblast-associated genes were further analyzed in existing breast cancer mRNA databases and through tissue array immunostaining. Using the sub-renal capsule graft model, fibroblasts from normal breast, DCIS and IDC tissues were co-transplanted with DCIS.com breast cancer cells.

**Results:**

Through transcriptome profiling, we found that DCIS fibroblasts were characterized by unique alterations in cell cycle and motility related genes such as PKMYT1, TGF-α, SFRP1 and SFRP2, which predicted increased cell growth and invasion by Ingenuity Pathway Analysis. Immunostaining analysis revealed corresponding increases in expression of stromal derived PKMYT1, TGF-α and corresponding decreases in expression of SFRP1 and SFRP2 in DCIS and IDC tissues. Grafting studies in mice revealed that DCIS fibroblasts enhanced breast cancer growth and invasion associated with arginase-1+ cell recruitment.

**Conclusion:**

DCIS fibroblasts are phenotypically distinct from normal breast and IDC fibroblasts, and play an important role in breast cancer growth, invasion, and recruitment of myeloid cells. These studies provide novel insight into the role of DCIS fibroblasts in breast cancer progression and identify some key biomarkers associated with DCIS progression to IDC, with important clinical implications.

## Introduction

As the most common form of pre-invasive breast cancer, ductal carcinoma *in situ* (DCIS) affects over 50,000 women in the US every year. DCIS is characterized by the growth of carcinoma cells confined within the breast ducts and is considered the immediate pre-cursor to invasive ductal carcinoma (IDC). DCIS is typically managed through a combination of lumpectomy and radiation therapy (1, 2). Despite standardized treatment, 8 to 25.5% of DCIS patients experience disease recurrence, often accompanied by IDC, indicating that a subset of patients tends to be under-treated. As the majority of DCIS cases do not progress to invasion, many patients may experience over-treatment and a reduced quality of life (3, 4). There are currently no reliable approaches to determine which cases of DCIS will progress to invasive disease. Low grade or small DCIS lesions may still become invasive (5–7). Moreover, some biomarkers such as Estrogen receptor (ER), HER2, Ki67, p16, and Cox2 are associated with disease recurrence but not progressive invasion (7, 8). By understanding the molecular and cellular processes associated with DCIS to IDC progression, we can identify new biomarkers to determine which cases of DCIS are more likely to progress to invasion and improve treatment of DCIS for patients.Fibroblasts are a major cellular component in the breast tumor microenvironment. The accumulation of fibroblasts in IDC is associated with disease progression and reduced survival (9, 10). Carcinoma associated fibroblasts (CAF) from IDC function differently than normal fibroblasts by significantly promoting growth, invasion, and anti-cancer drug resistance through secretion of soluble growth factors, cytokines, and extracellular matrix remodeling factors. The tumor promoting effects of IDC fibroblasts have been well documented (11, 12). While normal fibroblasts and CAFs express mesenchymal markers such as PDGFR-α and PDGFR-β, CAFs may be distinguished through increased expression of markers such as α-Smooth Muscle Actin (α -SMA) and FSP1. Transcriptome profiling and functional analysis suggest that distinct CAF populations regulate ECM remodeling, immunomodulation and M2 polarization or tumor proliferation and immunosuppression. These roles may be governed by different factors expressed in CAFs depending on molecular subtype of IDC (12–14). These studies demonstrate that CAFs have multiple, complex tumor promoting functions in breast cancer progression. Given the differences between normal breast fibroblasts and IDC fibroblasts, how did fibroblasts evolve over time to acquire these functions?

While the importance of fibroblasts in IDC is well established, less is known about the significance of fibroblasts in the progression of DCIS to IDC. Past studies indicate that fibroblasts could play an important role in DCIS progression. SPARC and SDC1 expression in fibroblastic cells have been detected in both DCIS and IDC (15). Increased expression of α-SMA in stroma is associated with high grade DCIS (16). Recent studies indicate that fibroblasts derived from DCIS have tumor promoting effects. Through 3D culture models, fibroblasts have been shown to regulate the growth and migration of human DCIS cells through an IL6 dependent mechanism (17). In animal models, HGF or CCL2 from fibroblasts regulate the transition of DCIS to IDC (18, 19). To date, the role of fibroblasts in DCIS progression remains poorly understood.

Here, we sought to determine whether DCIS fibroblasts were similar or distinct from normal fibroblasts and IDC fibroblasts at the transcriptome level using RNA seq, and likewise, determine the functional contributions of DCIS fibroblasts to breast cancer progression using a sub-renal transplant model. These studies provide novel insight into the functional significance of DCIS fibroblasts in breast cancer progression and identify biomarkers that could be translated into predictive biomarkers for DCIS.

## Materials and methods

### Ethical approval/informed consent details

The Human Research Protection Program at KUMC reviewed the study (#080193) involving the tissue collection for cell isolation and arrays. They determined that the investigators would not have access to patient identifiers and that the research posed minimal risk to subjects. As such the research was exempted from requiring IRB oversight. Tissue collection was facilitated by the Biospecimen Core Repository Facility (BCRF) is a facility approved by the Institutional Review Board to obtain written informed consent for tissue collection. The BCRF de-identified tissue samples prior to distribution. Existing medical records were used in compliance with KUMC and National Cancer Institute regulations. These regulations are aligned with the World Medical Association Declaration of Helsinki. Animals were maintained at KUMC in accordance with the Association for Assessment and Accreditation of Laboratory Animal Care. Animal experiments were performed under a protocol approved by the KUMC Institutional Animal Care and Use Committee and complied with the ARRIVE guidelines.

### Biospecimens

Tissue samples for fibroblast isolation were obtained from patients diagnosed with DCIS (n=6) or IDC (n=6) through surgical excision after collection of diagnostic samples. Status of ER, Progesterone Receptor (PR), HER2 and disease stage for these patient samples are summarized in **Supplemental Table 1**. Normal breast tissues were collected from patients undergoing reduction mammoplasty (n=2) or were indicated as normal adjacent breast (n=2) from patients with IDC.

For immunofluorescence staining, tissue array slides were obtained from the National Cancer Institute Cancer Diagnostics Program or the BCRF at KUMC, described in previous studies (20, 21). Briefly, tissue arrays from NCI contained 90 breast cancer core samples and 5 normal breast tissue cores, 3 fibroadenoma cores, and 20 control core samples comprised of prostate, colon and salivary gland and endometrium tissues. De-identified carcinoma or matching normal adjacent tissue samples were collected from patients who were diagnosed with breast cancer between 1985 and 1997, prior to adjuvant therapy. 85% of patients received adjuvant radiation, chemo- or hormone therapy, or a combination of therapies. BCRF tissue arrays contained 5 de-identified normal breast tissue samples and 32 core samples of stage I-IV breast ductal carcinoma in duplicate. When the patient datasets were combined, the average age of patients was 59 years, with an average follow-up time of 8.7 years.

### Cell isolation/culture

Fibroblasts were isolated from breast tissues using methods developed from (22) and modified in (23). This approach was used to isolate and characterize fibroblasts from mammary tissues in previous studies, demonstrating its reliability (19, 24, 25). Tissues approximately 1 cm^3^ in size, were digested overnight on ice in 10 ml of phosphate buffered saline (PBS) buffer containing: 5 mg/ml collagenase A (Sigma-Aldrich, cat no. 234153-1GM), 2 mg/ml trypsin (Sigma-Aldrich cat no. T3924-100ml), 1000 units/ml hyaluronidase (Sigma-Aldrich cat no. H3884) and 4 mg/ml DNase (Sigma-Aldrich cat no. D5025). Cells were pelleted, washed with PBS/10% FBS 3 times, and plated onto 10 cm dishes. The subsequent growth of cells was characterized as epithelial foci surrounded by fibroblastic cells. Fibroblasts were separated from epithelial cells through differential trypsinization in the following manner. The media was aspirated from tissue culture plates. Cells were washed in PBS and incubated in 1 ml of PBS containing 0.25% trypsin/0.54 mM EDTA at room temperature for 2 to 5 minutes, which allowed the more loosely adherent fibroblasts to lift off before epithelial cells. Fibroblasts were then transferred to a 15 ml conical tube containing 9 ml DMEM/10% FBS. Cells were pelleted and fibroblasts were transferred to new 10 cm dishes with DMEM/10% FBS containing penicillin/streptomycin. This procedure yielded approximately 100,000-250,0000 fibroblasts from tissues, with the cell number affected by the amount of adipose tissue in the patient samples provided. DCIS.com breast cancer cells originated from Dr. Fred Miller’s laboratory (26) and were cultured DMEM/10% FBS. All cell lines were maintained in culture no longer than 3 months at a time. Cells were tested after each freeze/thaw using the MycoAlert Mycoplasma Detection kit (Lonza cat no LT07).

### Immunocytochemistry

5000 fibroblasts were seeded per well in a 96 well plate in DMEM/10% FBS overnight. Cells were fixed in 10% neutral formalin buffer for 24 hours, permeabilized in methanol for 10 minutes at -20°C and blocked in PBS containing 3% FBS for 1 hour. Cells were then incubated with the following antibodies (1:100) for 24 hours in blocking buffer: anti-PDGFR-α (Cell Signaling Technology, cat no.5241), anti-FSP1 (Abcam, cat no. ab75550), α-SMA (Abcam cat no. 7817), anti-VEGFR2 (Santa Cruz Biotechnology, cat no.sc-393163) and anti-Pan-Cytokeratin (Biolegend, cat no.628602). Cells were washed in PBS 3 times and incubated with the following secondary antibodies (1:1000): anti-rabbit-biotinylated (Jackson Laboratories, cat no.111-065) to detect PDGFR-α or anti-mouse-biotinylated (Vector Laboratories, cat no.BA-9200) to detect VEGFR2, α-SMA or Pan-Cytokeratin. Biotinylated antibodies were incubated with streptavidin bound to horseradish peroxidase (HRP) (Vector Laboratories, cat no. SK4100) for 30 minutes. FSP1 was detected using secondary anti-rabbit-HRP (1:500, Avantor, cat no. 10150-732). Protein expression was detected through HRP reaction to 3, 3 -diaminobenzidine substrate (Vector Laboratories, cat no.SK4100).

### Sample preparation and whole transcriptome RNA sequencing

For RNA isolation, fibroblasts were grown in 10 cm dishes and expanded to 2 million cells in no more than 6 passages. After aspirating the media, cells were scraped and collected into Eppendorf tubes. RNA was isolated using the EZNA Total RNA kit I (Omega Biotech; cat no. R6834-02) according to manufacturer protocol. The RNA was subject to quality control analysis and whole transcriptome sequencing at the Genomics Core (University of Kansas Medical Center, Kansas City, KS). 500 ng total RNA was used for quality control analysis using the Agilent 2100 Bioanalyzer System. 500 ng total RNA was used to generate transcriptome libraries, using the TruSeq Stranded mRNA LT Sample Preparation Kit according to manufacturer’s protocol (Illumina; cat no.RS-122-2101/2102). Briefly, the total RNA fraction underwent oligo dT bead capture of mRNA, fragmentation, sizing, reverse transcription into cDNA and ligation with the appropriate indexed adaptors, followed by 13 cycles of library amplification. Final RNA-seq libraries were qPCR quantified using the Roche Lightcycler96 with FastStart Essential DNA Green Master (Roche; cat no.06402712001). The RNA-Seq libraries were adjusted to 4nM and pooled for multiplexed sequencing. Libraries were denatured and diluted to 12 pM (based on qPCR results) followed by clonal clustering onto the sequencing flow cell using the TruSeq Paired-End (PE) Cluster Kit v3-cBot-HS (Illumina; cat no.PE401-3001). The clonal clustering procedure was automated using the Illumina cBOT Cluster Station. The clustered flow cell was sequenced on the Illumina HiSeq 2500 Sequencing System using the TruSeq SBS Kit v3-HS (Illumina; cat no.FC401-3001). Following collection, sequence data was converted from.bcl file format to fastq files using bcl2fastq software and de-multiplexed into individual sequences for further downstream analysis. The fastq files were QC checked with FastQC (Babraham Institute). Reads were mapped against UCSC hg38 genome with STAR software (2.3.1z) and counted with HTSeq (0.5.4p3) (27, 28). Count tables were imported into R 3.1 and differential gene expression analysis was performed with *EdgeR* (3.12) (29). The resulting differential gene files were annotated with Ensembl’s biomaRt hsapiens_gene_ensembl ver84.

### RNA-Seq data analysis

Differentially expressed genes (DEG) were selected with a p-value<0.05 adjusted using the Benjamini-Hochberg false discovery rate and Log_2_ fold changes≥2, based on comparisons of: DCIS fibroblasts vs. normal breast fibroblasts, IDC fibroblasts vs. normal breast fibroblasts and DCIS fibroblasts vs. IDC fibroblasts. Principal component analysis (PCA) was conducted using the plotMDS function in *edgeR* using the default parameters: gene.selection=“pairwise”. Distances between samples on the plot represented the leading Log_2_ fold change, which was defined as the root-mean-square of the largest Log_2_ fold changes. DEGs were quantified and visualized using the online tools, VolcaNoseR (https://goedhart.shinyapps.io/VolcaNoseR/) and Venny ver 2.1 (https://bioinfogp.cnb.csic.es/tools/venny/). Matrix visualization and hierarchical clustering analysis were performed using Morpheus Broad-Institute on-line tool (https://software.broadinstitute.org/morpheus/). Gene regulatory networks, associated molecular and cellular functions and canonical pathways were investigated using Ingenuity Pathway Analysis (IPA) software (Ingenuity Systems, Qiagen). Networks were generated based on Ingenuity Knowledge Database. Predicted activation or inhibition of gene networks and upstream regulators was determined by absolute z-score ≥2. Additional information on specific downstream target genes were obtained from the Reactome database (https://reactome.org/)

### Semi-quantitative RT-PCR

Genes were amplified using primers summarized in **Supplemental Table 2**. The following PCR reaction mix was prepared in a 50 μl total volume: 1.2 μl (50-100 ng) of CDNA, 1x reaction buffer II (Applied Biosystems), 1.5 mM MgCl_2_, 0.2 mM dNTP, 1 μM individual primers, 0.5 μl (5 units) Amplitaq (Applied Biosystems). The CDNA were subject to the following PCR reaction using a Biorad ThermoCycler: initial denaturation at 94°C for 5 minutes, followed by 30 cycles of denaturation at 94°C for 45 sec, primer annealing at 60°C for 45 sec and primer extension of 72°C for 2 minutes. Reactions were resolved on a 1% agarose gel with 1 kB ladder.

### Identification of established mRNA stromal breast cancer datasets

A candidate search for existing mRNA stromal breast cancer datasets was performed in Gene expression Omnibus (https://www.ncbi.nlm.nih.gov/geo/) using the search terms: “breast cancer associated fibroblast, normal breast fibroblast, breast cancer stroma, DCIS stroma or normal breast stroma” on January and November 2022. Studies were excluded that involved lobular breast cancer, which lacked normal breast or normal stroma, or were genetically manipulated or stimulated with recombinant proteins or drugs. 5 data sets analyzed are summarized in **Supplemental Table 3**.

### Subrenal graft

IL2Rgamma-/-NOD SCID mice, 6-8 weeks of age were obtained from Jackson Laboratories. Co-transplantation of fibroblasts with DCIS.com cells were performed using methods previously described (30). Briefly, fibroblasts from individual patient samples were expanded in culture. 250,000 fibroblasts from an individual case were embedded with or without 100,000 carcinoma cells in 50 μl of type I collagen overnight. Mice were anesthetized using 2% isoflurane. A Y incision was made in the back, 1 cm from the tail to expose the left or right kidney. Using spring scissors, a 1-2 mm pocket was made into the subrenal capsule. One collagen plug containing cells was inserted into the pocket. Wounds were closed using gut absorbable sutures. Animals were monitored and palpated twice weekly for tumor formation. Tumors were measured by caliper. Mice were sacrificed 21 days post-transplantation. Animals were maintained under an approved IACUC protocol in AALAAC accredited facilities.

### Scoring of tumor invasion

Tumors stained with phalloidin and pan cytokeratin were scored using procedures described in previous studies (19). Briefly, tissues were sectioned at 3 different depths approximately 50 microns apart, imaged at 10x magnification, and blindly scored. 1 = border between tumor and kidney is well defined. 2= some tumor cells are present in kidney tissue; border is less defined. 3= extensive tumor cell invasion is observed into the kidney parenchymal; border between tumor and kidney is undefined. Percent invasiveness of grafts was determined by the total number of sections scored per sample.

### Immunohistochemistry/Immunofluorescence staining

Tissues were fixed in 10% neutral formalin buffer for 24 hours and processed into paraffin using procedures previously described (31). 5-micron sections were de-waxed, processed through a series of 2 changes of xylenes and 1 change each of 100%, 95%, 70% and 50% ethanol and then washed in PBS.

#### Human breast tissues

After PBS washing, slides underwent antigen retrieval through low pressure cooking in 10 mM Tris/EDTA pH 9.0/0.5% Tween-20 for 3 minutes. Sections were permeabilized with PBS/10% Methanol for 10 minutes. Slides were blocked in PBS containing 3% Fetal bovine serum for 1 hour. Slides were incubated with antibodies (1:100) to: SFRP1 (Cell Signaling Technology, cat no. 3534), SFRP2 (Santa Cruz Biotechnology, cat no. sc365524), PKMYT1 (Cell Signaling Technology, cat no.4282), TGF-α (Santa Cruz Biotechnology, cat no. 374433), Collagen IVα1 (Novus Biologicals, cat no. NB1206586, PDGFR-α (Cell Signaling Technology, cat no.5241), α-SMA (Spring Biosciences, cat no. M4712) or CD34 (Novus Biologicals, cat no. NBP2-32932) overnight in PBS/3%FBS. SRFP1 was detected by donkey anti-rabbit-Alexa-Fluor-647 (Invitrogen, cat no. A31573). SFRP2 and PKMYT1, TGF-α were detected by anti-mouse-Alexa-Fluor-568 (Invitrogen, cat no. A10037). PDGFR-α, Collagen A4, and α-SMA were detected by anti-rabbit-biotinylated antibodies. CD34 was detected by anti-mouse-biotinylated antibodies. This was followed by incubation with streptavidin-alexa-fluor-488 (Invitrogen, cat no. S11223) for 30 minutes. After washing in PBS, slides were counterstained with DAPI and mounted with 1:1: PBS: glycerol.

#### Subrenal graft tissues

Pan cytokeratin/phalloidin staining was performed using procedures previously described (19). For arginase-I, slides underwent antigen retrieval through low pressure cooking in 10 mM sodium citrate pH 6.8.0/0.5% Tween-20 for 3 minutes. Peroxidases were quenched for 30 minutes in PBS containing 3% H202 and 10% Methanol and blocked with PBS/3% FBS for 1 hour. Slides were incubated with antibodies (1:100) to arginase-I (Cell Signaling Technology, cat no. 93668) overnight in blocking buffer. Slides were incubated with appropriate biotinylated antibodies (1:1000) for two hours, followed by streptavidin-peroxidase (cat no. PK-6100, Vector Laboratories, Burlingame, CA) and incubation with DAB substrate. Slides were counterstained with Mayer’s hematoxylin, dehydrated and mounted.

### Image quantification

Images were acquired at 10x magnification using a FL-Auto EVOS Imaging System. Software analysis of biomarker expression in breast tissues was performed adapting methods previously (20). Briefly, images were imported into Image J. Images were opened in Image J software (NIH) and converted to greyscale. Background pixels resulting from luminosity of brightfield images were removed by threshold adjustment. Tumor epithelium was distinguished from the stroma by differences in nuclear and cellular morphology, and tissue architecture. To select for specific tissues, the lasso tool was to select and crop out stroma or epithelium.” Images were the subject to particle analysis. Staining and total areas were expressed as particle area values of arbitrary units. Positive biomarker staining values were normalized total stromal or epithelial values identified by DAPI staining.

### Statistical analysis

For IPA analysis of RNA seq data, statistical analysis was performed using Fisher’s exact test, which calculated the probability of random associations between molecules and molecular/cellular function or canonical pathway. For all other data, statistical analyses were performed using GraphPad Prism Software ver 9.0. To determine whether parametric or non-parametric tests were used, Kolmogorov-Smirnov test of normality was performed. The combined KUMC and NCI patient tissue cohorts were observed to have a non-normal distribution and were uneven due to two factors. Information on prognostic factors was not provided for all patients from either cohort. In addition, some tissue samples did not adhere to the slide during staining. As such, protein expression values and their relationships to clinical data were analyzed using non-parametric Kruskal-Wallis test with Dunn’s *post-hoc* comparison, Mann-Whitney test, or Spearman Correlation test. Due to small, uneven sample size and non-normal distribution of sub-renal graft data, Kruskal Wallis test with Dunn’s *post-hoc* comparison was used. Uneven distribution of subrenal graft data was due to varying number of fibroblasts isolated from tissues, which affected the number of potential grafts. Statistical significance was determined by p<0.05 or –Log(p value)≥1.3.

## Results

### Transcriptome profiling reveals significant differences among normal, DCIS and IDC fibroblasts

To determine potential similarities or differences of breast fibroblasts from different stages of disease progression, fibroblasts were first isolated from normal breast tissue, DCIS and IDC tissues using procedures described (23). We noted that fibroblasts were isolated from DCIS and IDC tissues with differences in ER, PR and HER2 expression. IDC tissues were from various stages of disease (**Supplemental Table 1**). Purity of fibroblasts in culture was determined through immunocytochemistry staining for positive expression of fibroblastic markers (PGDFR-α, FSP1, α-SMA) and for the absence of the epithelial marker (Pan-Cytokeratin) and the endothelial marker (VEGFR2) (**Supplemental Figure 1**). We then characterized the gene expression profiles of breast fibroblasts using RNA seq. The sequencing data were analyzed in a multi-stepwise workflow (**Supplemental Figure 2**).To examine the similarities and differences of fibroblasts from normal breast, DCIS, and IDC tissues, PCA was performed using the RNA seq data (**Supplemental Figure 3**). PCA revealed that there was variation among the types of fibroblasts, and that not one type was fully distinct. 3/4 cases of normal fibroblasts (9019, 8727, 4009), 5/6 cases of DCIS fibroblasts (301413, 12115, 80H, 1213249) and 3/6 cases of IDC fibroblasts (8727, 9019, 2300) were close together in 2D. The other 3 cases of IDC fibroblasts (8870, 2760, 8661) and the other case of normal fibroblasts (2525) were more distinctly separated. The other case of DCIS fibroblasts (21714) was also distinct from the other fibroblast samples. These data indicated heterogeneity within fibroblast groups, particularly with normal breast and IDC fibroblasts. These data have been included as **Supplemental Figure 3**.

**Figure 1:**
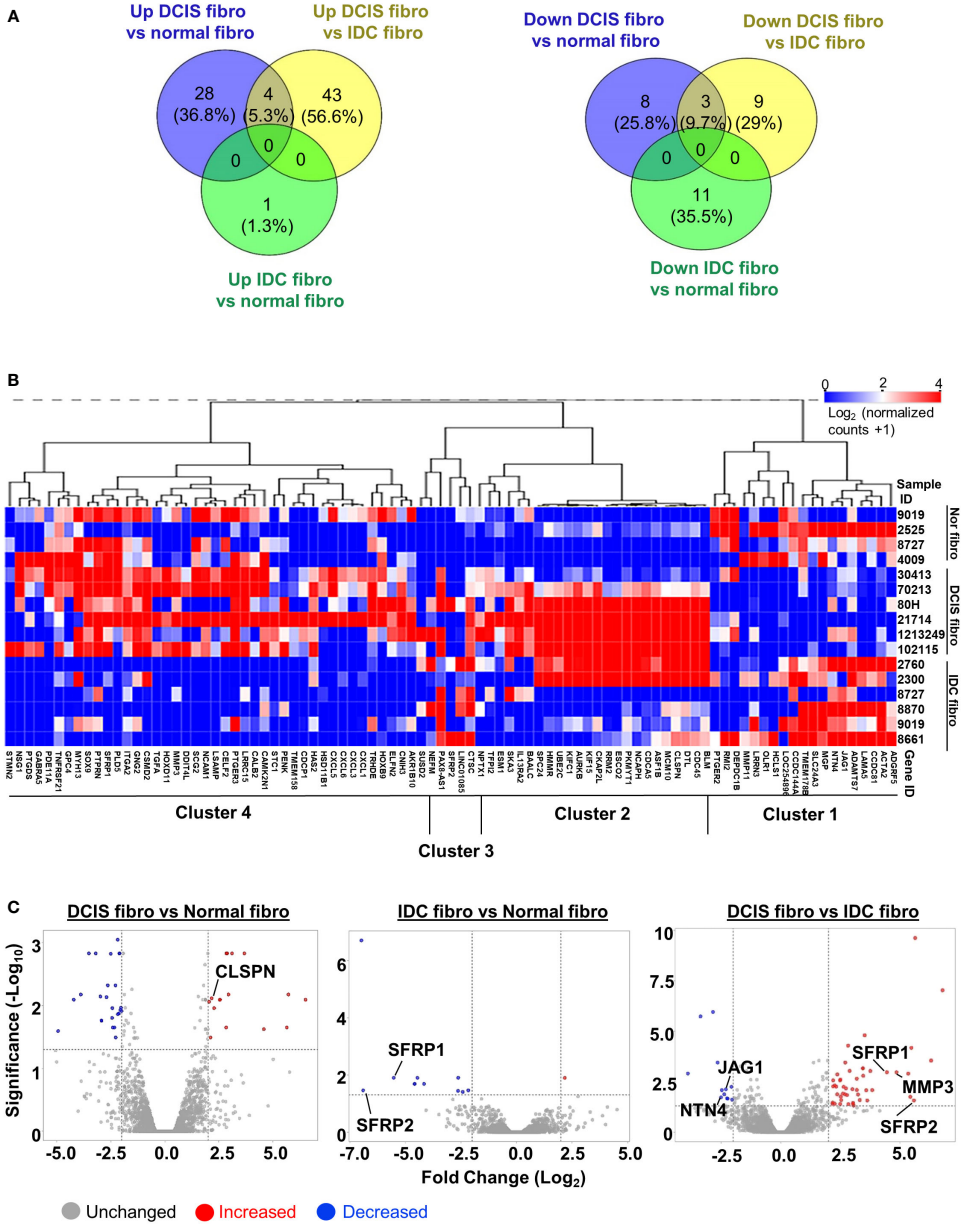
RNA seq profile of differentially expressed genes in breast fibroblasts. **(A)** Venn diagram of upregulated or downregulated genes in: DCIS fibroblasts vs. normal fibroblasts (NF) DCIS fibroblasts vs. IDC fibroblasts and IDC fibroblasts vs. normal breast fibroblasts (Fold Change (Log_2_) ≥2, FDR adjusted p<0.05). **(B)** Hierarchical clustering analysis of upregulated and downregulated genes identified through differential comparisons of fibroblast groups. **(C)** Volcano plot of upregulated or downregulated genes in: DCIS fibroblasts vs. normal fibroblasts, DCIS fibroblasts vs. IDC fibroblasts and IDC fibroblasts vs. normal fibroblasts. Red dots represent upregulated genes. Blue dots represent downregulated genes. X -axis is defined by the -Log_10_ FDR adjusted p-value ≥1.3. Y-axis is defined by Fold Change (Log_2_) ≥2. DEGs that were chosen for validation by RT-PCR are labeled on plots.

We then quantified the number of differentially expressed genes (DEGs) through comparisons of DCIS fibroblasts vs. normal fibroblasts, IDC fibroblasts vs. normal breast fibroblasts and DCIS fibroblasts vs. IDC fibroblasts. Genes were selected based on p<0.05 adjusted with Benjamini-Hochberg FDR and Fold change (Log_2_) ≥2. DCIS fibroblasts showed an upregulation of 28 genes and downregulation of 8 genes, compared to normal fibroblasts (**Figure 1A**). In IDC fibroblasts, 1 gene was upregulated, and 11 genes were downregulated, compared to normal fibroblasts. Compared to IDC fibroblasts, DCIS fibroblasts showed an upregulation of 43 genes and downregulation of 9 genes. There were 4 overlapping genes that were upregulated in DCIS fibroblasts in comparison with normal and IDC fibroblasts. Hierarchical clustering analysis was performed on 91 genes identified through differential comparisons of fibroblast groups (**Figure 1B**). Four primary clusters of genes were observed. Cluster 1 genes were lowly expressed in DCIS fibroblasts and more highly expressed in normal and IDC fibroblasts. Cluster 2 genes were highly expressed in 5/6 DCIS fibroblasts and more lowly expressed in normal fibroblasts and 4/6 IDC fibroblasts. IDC fibroblasts 2760 and 2300 deviated from other IDC fibroblasts with their expression of cluster 2 genes. Cluster 3 genes were highly expressed in DCIS and IDC fibroblasts, and more lowly expressed in normal fibroblasts. Cluster 4 genes were lowly expressed in IDC fibroblasts and more highly expressed in normal and DCIS fibroblasts. Overall, we detected a significant number of differentially expressed genes that can distinguish normal, DCIS, and IDC fibroblasts from one another.

To validate integrity of RNA seq data, semi-quantitative RT-PCR was conducted on non-overlapping genes (CLSPN, JAG1, NTN4, MMP3) and overlapping genes (SFRP1, SFRP2) identified through volcano plots (**Figure 1C**; **Supplemental Figure 4**). CLSPN was upregulated in DCIS fibroblasts vs. normal fibroblasts. SFRP1 and SFRP2 were downregulated in IDC fibroblasts vs. normal fibroblasts and upregulated in DCIS fibroblasts vs. IDC fibroblasts. In DCIS fibroblasts vs. IDC fibroblasts, MMP3, SFRP1 and SFRP2 were upregulated while NTN4 and JAG1 were downregulated (**Supplemental Figure 4**). In summary, significant differences in gene expression were detected among normal, DCIS and IDC fibroblasts.

### Cell growth, cell cycle and DNA damage related genes distinguish DCIS fibroblasts from normal fibroblasts

IPA software was used to classify DEGs and characterize the upstream regulatory networks, canonical signaling pathways and predicted cellular functions associated with DEGs identified through comparisons of fibroblast groups. The overall goal of IPA analysis was to understand the molecules distinguishing DCIS fibroblasts from normal breast fibroblasts and IDC fibroblasts. Genes selected for analysis were based on p-values adjusted for Benjamini-Hochberg FDR and Fold change (Log_2_)≥2. IPA classified genes according to molecular and cellular functions, allowing us to understand what categories of genes were changed between fibroblast groups. Upstream regulator analysis was then performed to generate networks that diagramed relationships between predicted upstream regulators with identified downstream gene targets. Upregulated downstream targets were indicated by red; downregulated gene targets were indicated by green. Predicted activation and inhibitory relationships were indicated by orange or blue lines and allowed us to determine how consistent the actual measurements of target genes were to the predicted relationships between upstream regulator and downstream gene targets. Significant activation was indicated by upstream regulators was indicated by z-score≥2. Z-scores <-2 predicted significant inhibition. From these gene networks, canonical signaling pathways were identified, allowing us to understand the coordinated functions of DEGs. Statistically significant pathways were identified by -Log(p-value)≥1.3. Finally, the gene networks allowed us to predict cellular functions, providing insight into the potential functional significance of DEGs in fibroblasts in cancer. Statistical significance of predicted cellular functions was determined by z-score.

**Figure 2:**
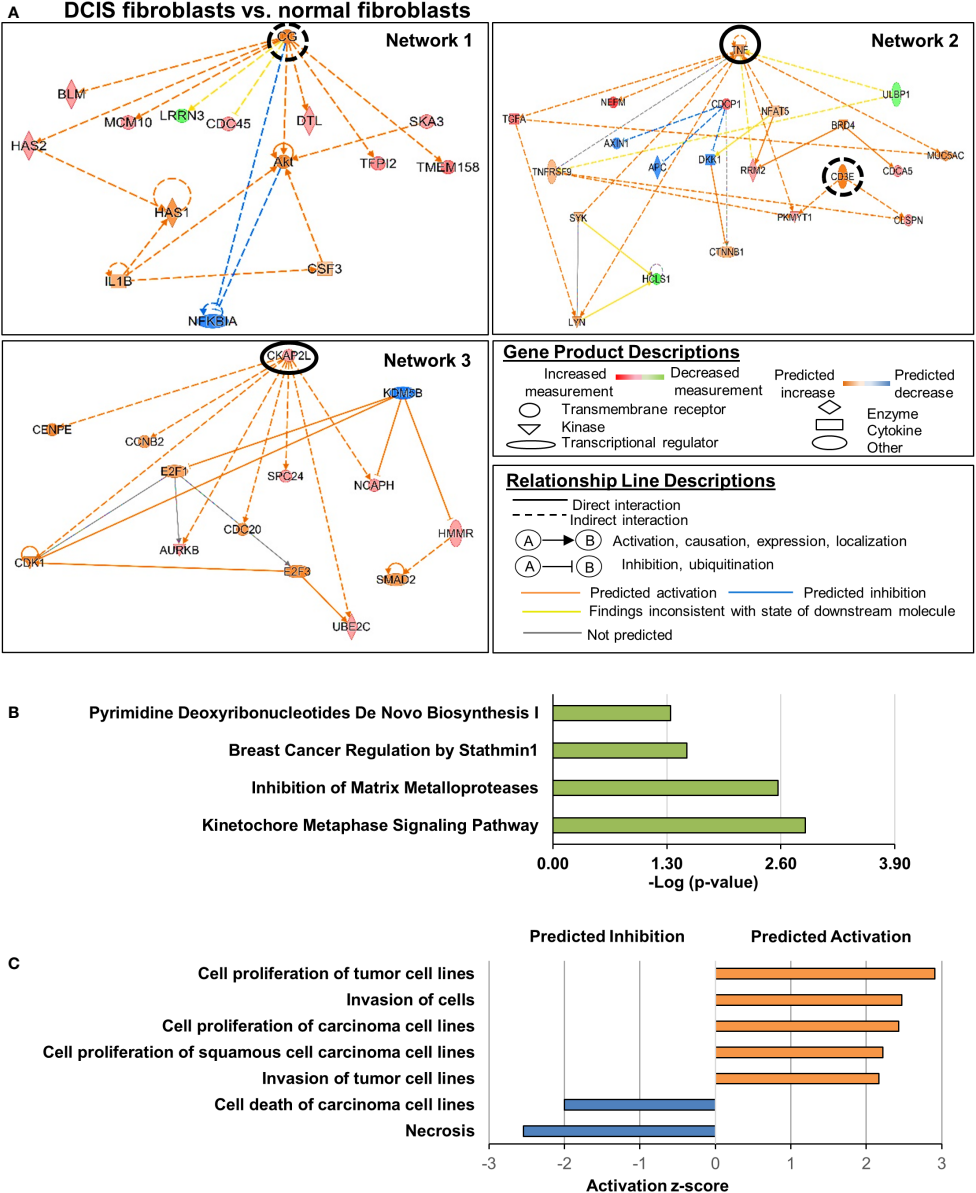
Cell growth and invasion related genes altered in DCIS fibroblasts compared to normal fibroblasts. Genes were selected showing a Log_2_ Fold Change >2 with FDR adjusted p-value <0.05. **(A)** Gene regulatory networks altered in DCIS fibroblasts. Upstream regulators of interest are circled. A solid black circle indicates z-score ≥2, and a dashed black circle indicates z-score <2. Overlapping p-value of the upstream regulators range from 1.56e-7 (Network 1), 1e-6 to 1.67e-6 for Network 2 and 4.7e-6 for Network 3. **(B)** Significant canonical pathways altered in DCIS fibroblasts vs. normal fibroblasts. **(C)** Predicted activation or inhibition of cellular functions. Absolute z-score ≥2 was used to determine significant activation or inhibition in panels **(A, C)** -Log (p-value) ≥1.3 was used to determine statistical significance in panel **(B)**.

We first analyzed DEGs in DCIS fibroblasts vs. normal fibroblasts. There were significant alterations in genes categorized under: cell cycle, cellular assembly and organization, cell death and survival, cellular development, and cellular growth and proliferation (**Table 1**). We next examined the upstream regulatory networks; three were identified (**Figure 2A**). In network 1, the primary predicted upstream regulator was Choriogonadotropin (CG) complex whose downstream targets DNA replication genes: BLM RecQ helicase, MCM10 and CDC45, which were upregulated. Other CG associated downstream targets included: Hyaluronan synthases 1 and 2 (HAS1, HAS2), the E3 Ubiquitin Protein Ligase Homolog gene DTL, the serine protease inhibitor gene TFIP2, and the transmembrane protein and growth regulator gene TMEM158, which were also upregulated. Leucine Rich Repeat Neuronal 2 (LRRN2), a cell adhesion and signal transduction gene, was downregulated. Network 2 was primarily regulated by TNF and CD3E. TNF downstream targets involved upregulation of cell growth, DNA replication and cell growth related genes: transforming growth factor alpha (TGFA), NEFM, RRM2, PKMYT1, and MUC5AC while downregulating ULBP1, which participates in DNA replication and cell growth. CD3E-mediated downstream targets included DNA damage response and DNA replication genes, PKMYT1 and CLSPN. Network 3 included gene targets related to kinetochore assembly, cell cycle and DNA replication: AURKB, SPC24, NCAPH, HMMR and UBEC2C, which were regulated by CKAP2L, a mitotic spindle protein gene. In all three networks, the increased expression of downstream genes was consistent with their predicted activation. These genes encoded intracellular proteins with the exceptions of TGFA, which encoded a soluble growth factor, and TMEM158, which encoded a transmembrane protein. Canonical signaling pathway analysis revealed that these genes were associated with pyrimidine deoxyribonucleotide *de novo* biosynthesis, breast cancer regulation by Stathmin1, inhibition of matrix metalloproteinases, and kinetochore metaphase signaling pathway (**Figure 2B**). Lastly, z-score analysis of the gene expression changes in DCIS fibroblasts predicted activation of cell proliferation and invasion along with inhibition of cell death (**Figure 2C**). In summary, DCIS fibroblasts were distinguished from normal fibroblasts through increased expression of genes related to DNA damage, DNA replication, and cell growth.

**Table 1:** IPA classification of genes according to molecular and cellular function.

Cellular Function	Fibroblast Group Comparisons					
	DCIS fibroblast vs Normal fibroblast		ICC fibroblast vs Normal fibroblast		DCIS fibroblast vs IDC fibroblast	
	p-value range	No.molecules	p-value range	No.molecules	p-value range	Overlap
Cell cycle	2.79E-02-7.04E-09	17				
Cellular assembly and organization	2.99E-02-8.31E-05	13				
Cell death and survival	2.67E-02-4.66E-05	14	3.02E-02-5.77E-04	7		
Cellular development	2.79E-02-3.10E-06	15	3.02E-02-5.77E-04	4	3.71E-02-1.40E-06	21
Cellular growth and proliteration	2.79E-02-3.10E-06	13			3.71E-02-1.54E-04	19
Cellular movement			2.11E-02-1.54E-03	5	3.91E-02-0.19E-08	25
Cell-To-Cell Signaling and Interaction			2.88E-03-5.77E-04	2	3.96E-02-1.33E-07	19
Cell Signaling			1.77E-02-1.09E-03	2		
Cell function and maintenance					3.71E-02-1.95E-03	13

Genes selected for IPA were based on Fold Change (Log_2_) ≥2, FDR <0.05 determined through differential comparison of fibroblast groups. Statistical analysis in IPA was performed using Fisher’s exact test. Statistical significance was determined by p-value <0.05.

Interestingly, while DCIS fibroblasts showed many changes in gene expression, IDC fibroblasts showed fewer changes when compared to normal fibroblasts. These genes were categorized under cell death and survival, cellular development, cellular movement, cell-to-cell signaling and interaction and cell signaling (**Table 1**). One gene regulatory network was identified with four upstream regulators, which were associated with gene targets that were downregulated (**Supplemental Figures 5A, B**). Notably, the upstream regulators TGFB1 and BMP2 growth factors were associated with both NOG, a secreted polypeptide that binds and inactivates TGF-β signaling proteins, and SFRP1, a soluble factor t binds and inactivates WNT signaling proteins. In addition, the transcriptional regulator NEUROG1 was associated with NOG and Secretogrannin2 (SCG2), a secreted chemotactic peptide. The transcriptional regulator POU5F1 was associated with the downstream targets Neural Cell Adhesion Molecule1 (NCAM1) and Tumor Necrosis Factor Receptor Superfamily 21 (TNFRSF21), which induces apoptosis. In this network, the increased or decreased expression of downstream genes were consistent with their predicted activation or inhibition state. These downstream target genes were associated with canonical pathways including: WNT/β−catenin signaling, prostanoid biosynthesis, choline biosynthesis, phospholipases, and eicosanoid signaling (**Supplemental Figure 5C**). By z-score analysis, there were no significant cellular functions predicted with these genes. In summary, IDC fibroblasts were distinguished from normal fibroblasts through their downregulation of genes that negatively regulate cell death, movement, development and signaling.

### Cell movement and invasion related genes distinguish DCIS fibroblasts from IDC fibroblasts

**Figure 3:**
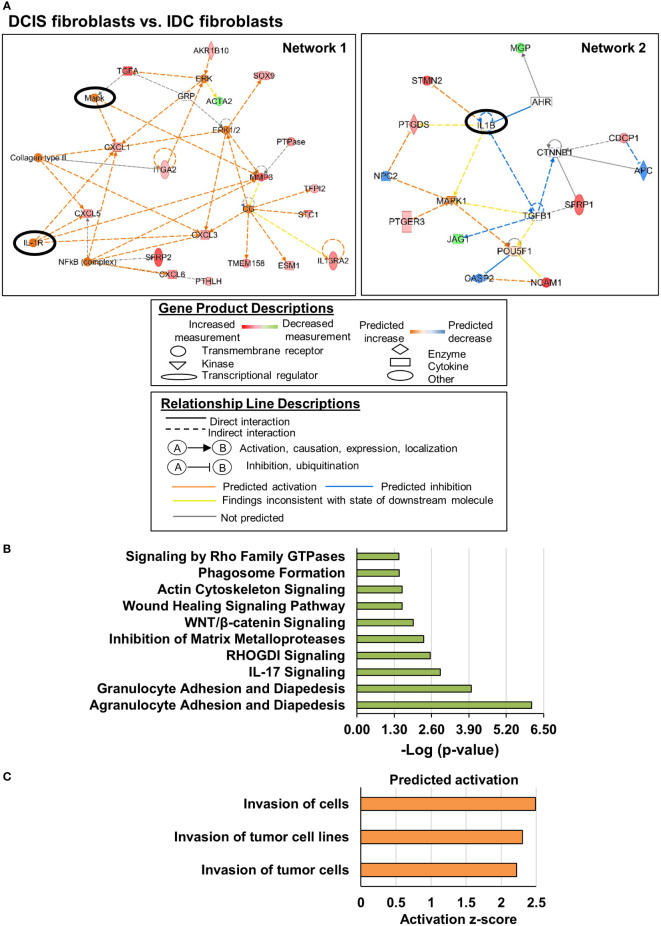
Invasion related genes are altered in DCIS fibroblasts compared to IDC fibroblasts. Genes were selected showing a Log_2_ Fold Change >2 with FDR adjusted p-value < 0.05. **(A)** Gene regulatory networks are altered in DCIS fibroblasts. Upstream regulators of interest are circled. A solid black circle indicates z-score ≥2. **(B)** Significant canonical pathways altered in DCIS fibroblasts vs. IDC fibroblasts Overlapping p-value of the upstream regulators range from 3.53e-6 to 5.35e-9 for Network 1 and 7.5e-7 for Network 2. **(C)**. Predicted activation or inhibition of cellular functions Absolute z-score ≥2 was used to determine significant activation or inhibition in panels **(A, C)** -Log (p-value) ≥1.3 was used to determine statistical significance in panel **(B)**.

Lastly, we examined the functional roles and mechanistic networks associated with gene expression changes in DCIS fibroblasts compared to IDC fibroblasts. There were significant alterations in genes categorized under: cellular development, cellular growth and proliferation, cellular movement, cell-to-cell signaling and interaction, and cell function and maintenance (**Table 1**). Two upstream regulatory networks were characterized in DCIS fibroblasts vs. IDC fibroblasts (**Figure 3A**). In network 1, significant upstream regulators included: MAPK kinase ERK1/2 and interleukin receptor IL1R. Associated downstream targets of ERK1/2 included the chemokines CXCL1, CXCL3, CXCL5, and CXCL6 and interleukin receptor IL13RA2, which were all observed to be upregulated in DCIS fibroblasts relative to IDC fibroblasts. Associated downstream targets of IL1R included: CXCL1, CXCL3, CXCL5 and matrix metalloproteinase gene MMP3, which were all upregulated as well. In this network, the increased expression of downstream genes was consistent with their predicted activation. In network 2, IL1β was the significant upstream regulator. Downstream targets included Prostaglandin D2 Synthase (PTGDS), and microtubule destabilizer gene Stathmin2 (STMN2), which were upregulated. JAG1, a downregulated gene target and SFRP1, an upregulated gene target appeared to be indirectly related to IL1-β through a predicted inhibition of TGF-β1. In both networks, many of the identified genes encoded transmembrane or extracellular proteins such as: NCAM1, CXCL1, CXCL3, CXCL5, CXCL6, SFRP1 and SFRP2. All other genes encoded intracellular proteins. Canonical signaling pathway analysis revealed that these genes were associated with granulocyte and agranulocyte adhesion and diapedesis, IL17 signaling, RHO and RHOGDI signaling, inhibition of matrix metalloproteinases, WNT/β-catenin signaling, wound healing, actin cytoskeleton signaling, and phagosome formation (**Figure 3B**). Z-score analysis predicted increased cell invasion (**Figure 3C**). In summary, DCIS fibroblasts were distinguished from IDC fibroblasts through alterations in expression of cell movement and cell invasion-related genes.

### Comparison of data with existing mRNA breast cancer stromal datasets

Previous studies have profiled the transcriptome of whole stroma from normal breast, DCIS, or IDC tissues and the transcriptome of fibroblasts from normal breast or IDC tissues (**Supplemental Table 3**). We compared our experimental dataset with these mRNA datasets obtained from Gene Expression Omnibus. We first probed for expression of 15 gene targets from our experimental dataset, with 5 gene targets from each fibroblast comparison group (**Supplemental Table 4**). Of the 5 datasets examined, only the GSE9104 dataset generated by Finak et al. showed some agreement with the experimental dataset. The GSE9104 dataset revealed downregulation of SFRP1, TNFRSF21, and TMEM158 in IDC stroma vs. normal stroma, consistent with downregulation of these genes in IDC fibroblasts (**Supplemental Table 4**). We then probed our experimental dataset for expression of significant stromal gene signatures identified from these published datasets (32–36). However, we did not find significant agreement of stromal gene expression in pre-existing datasets with our experimental dataset. In summary, there were significant differences between the experimental dataset and pre-existing mRNA datasets.

### Validation of PKMYT1, TGF-α, SFRP1 and SFRP1 expression through immunohistochemistry

To further validate gene targets from the experimental dataset, immunohistochemistry was performed on normal breast, DCIS, and IDC breast tissues. Gene targets included PKMYT1 and TGFA, which are cell growth and cell cycle-related genes upregulated in DCIS fibroblasts vs. normal fibroblasts. We also examined expression of SFRP1 and SFRP2, which were cell differentiation and cell survival-related genes upregulated in DCIS fibroblasts vs. IDC fibroblasts but also downregulated in IDC fibroblasts vs. normal breast fibroblasts. Protein expression was quantified using an Image J approach validated in previous studies (20, 21). This approach allowed us to distinguish biomarker expression in the stroma and epithelium across the different breast tissue groups.

**Figure 4:**
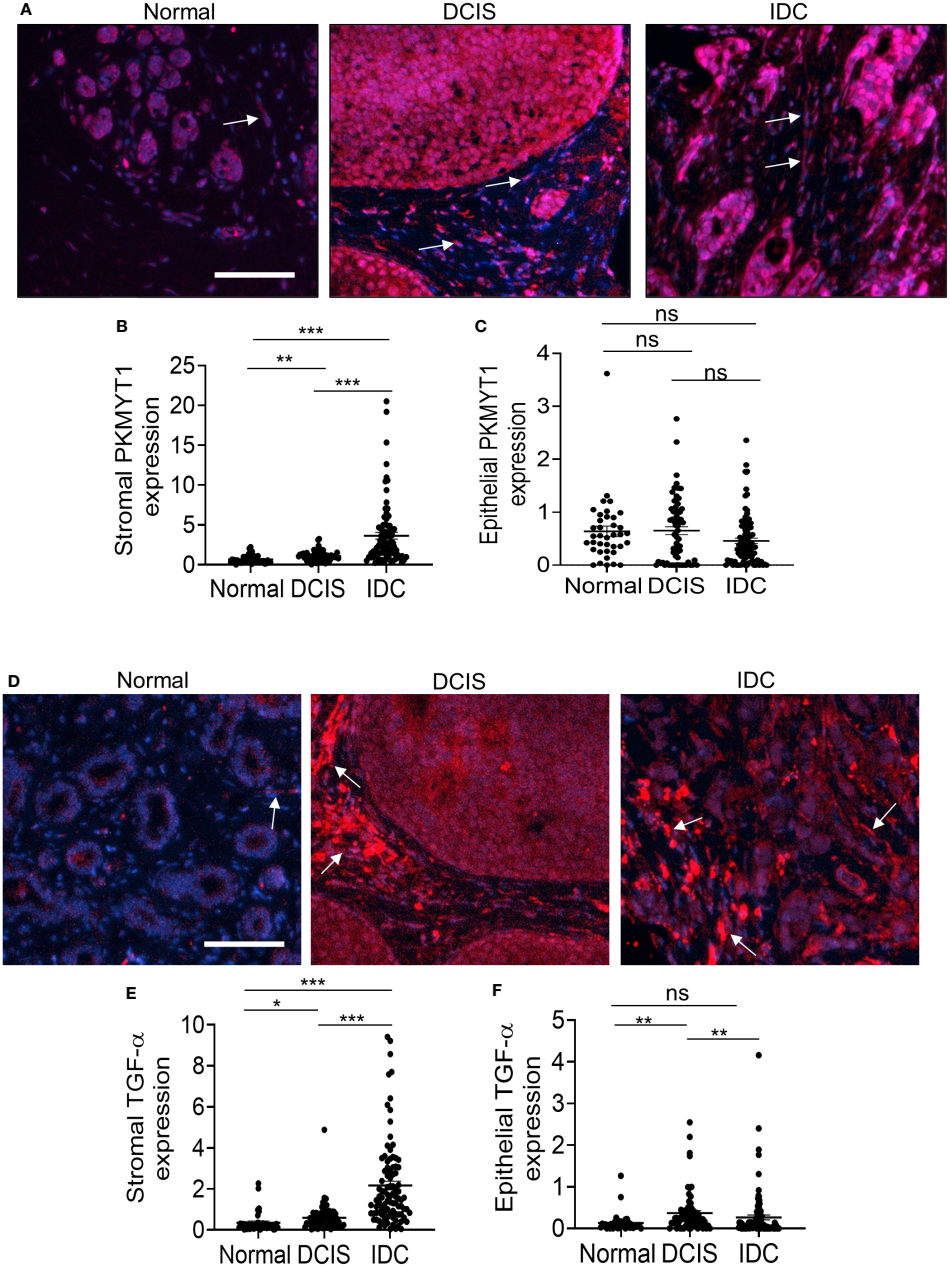
PKMYT1 and TGF-α protein expression are elevated in DCIS and IDC stroma. Normal adjacent breast to DCIS (n=38), DCIS (68) or IDC (n=95) were immunofluorescence stained for expression of PKMYT1 or TGF-α. Total and tissue specific expression were quantified by Image J. Mean ± SEM are shown. **(A)** PKMYT1, representative staining shown with DAPI counterstain. **(B)** Stromal PKMYT1 expression. **(C)** Epithelial PKMYT1 expression. **(D)** Representative TGF-α expression with DAPI counterstain. **(E)** Stromal TGF-α expression. **(F)** Epithelial TGF-α expression. Arrows point to positive staining in the stroma. Scale bar=100 microns. Expression was quantified by Image J. Statistical analysis was determined by Kruskal Wallis test with Dunn’s *post-hoc* comparison. Statistical significance was determined by p<0.05. *p<0.05, **p<0.01, ***p<0.001, ns= not significant.

We first examined expression patterns of PKMYT1 and TGF-α. PKMYT1 was detected in both breast stroma and epithelium. There were significant increases in stromal expression of PKMYT1 in DCIS and IDC compared to normal breast stroma, with the highest level of expression detected in IDC stroma (**Figures 4A, B**). There were no significant differences in epithelial PKMYT1 expression among normal breast, DCIS, and IDC tissues (**Figure 4C**). TGF-α expression was detected more strongly in the breast stroma than the epithelium. In the stroma, TGF-α was most highly expressed in IDC compared to DCIS and normal breast tissues (**Figures 4D, E**). In the epithelium, TGF-α was more highly expressed in DCIS than in IDC tissues (**Figure 4F**). In summary, PKMYT1 and TGF-α were significantly expressed in DCIS and IDC stroma.

**Figure 5:**
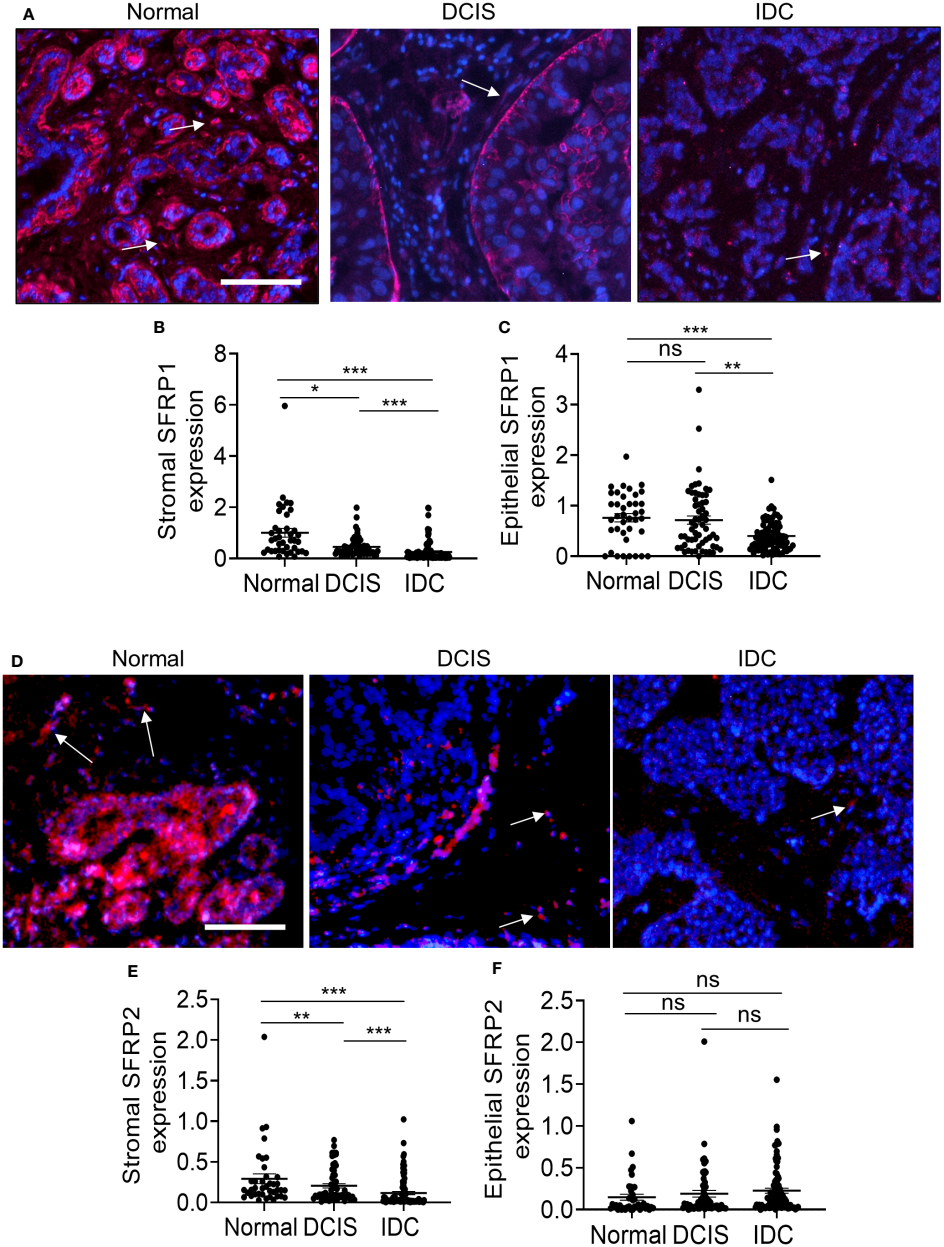
SFRP1 and SFRP2 protein expression are downregulated in DCIS and IDC stroma. Normal adjacent breast to DCIS (n=67), DCIS (79) or IDC (n=135) were immunofluorescence stained for expression to SFRP1 or SFRP2. Expression was quantified by Image J (arbitrary units). Mean ± SEM are shown. **(A)** SFRP1, representative staining shown with DAPI counterstain. **(B)** Stromal SFRP1 expression. **(C)** Epithelial SFRP1 expression. **(D)** Representative SFRP2 expression with DAPI counterstain. **(E)** Stromal SFRP2 expression. **(F)** Epithelial SFRP2 expression. Arrows point to positive staining in the stroma. Scale bar=100 microns. Statistical analysis was determined by Kruskal Wallis test with Dunn’s *post-hoc* comparison. Statistical significance was determined by p<0.05. *p<0.05, **p<0.01, ***p<0.001, ns= not significant.

Next, we examined SFRP1 and SFRP2 protein levels. SFRP1 expression was strongly detected in normal breast tissues in the stroma and epithelium (**Figure 5A**). Decreased SFRP1 expression in the stroma was observed in both DCIS and IDC. Decreased SFRP1 expression in the epithelium was observed in IDC but not DCIS tissues (**Figures 5B, C**). SFRP2 was also strongly detected in normal breast stroma and epithelium (**Figure 5D**). Like SFRP1, decreased SFRP2 expression in the stroma was observed in both DCIS and IDC. In contrast to epithelial SFRP1, epithelial SFRP2 did not show any significant changes in expression among groups (**Figure 5E, F**). In summary, SFRP1 and SFRP2 were significantly reduced in DCIS and IDC stroma.

We evaluated the potential clinical significance of stromal PKMYT1, TGF-α, SFRP1 and SFRP2 expression by examining for their associations with commonly assessed prognostic factors and clinical outcome. In DCIS, stromal PKMYT1 expression correlated with ER expression and inversely correlated with HER2 expression. Stromal SFRP2 inversely correlated with PR and HER2 expression and positively associated with Ki67 expression (**Supplemental Figure 6A**). In IDC, there were no significant associations of biomarker expression with prognostic factors (**Supplemental Figure 6B**). Previous studies had demonstrated that CCL2 was expressed in DCIS and IDC stromal tissues, and that stromal derived CCL2 was associated with invasive progression and disease recurrence (19, 20, 37). Here, we determined whether stromal CCL2 was associated with the other stromal markers. We found that stromal CCL2 positively associated with stromal SFRP2 expression in DCIS but not IDC (**Supplemental Figures 6A, B**). Lastly, we determined whether the stromal markers were associated with vital status of breast cancer patients, defined by alive vs. dead. Of the biomarkers examined, only SFRP1 expression in stroma showed an association with vital status. SFRP1 in breast cancer stroma tended to be lower in patients who were alive at this follow-up time (**Supplemental Figure 7**). These data indicate that stromal PKMYT1 and SFRP2 were associated with some prognostic factors. Furthermore, lower SFRP1 in IDC stroma was associated with survival independent of known prognostic factors.

**Figure 6:**
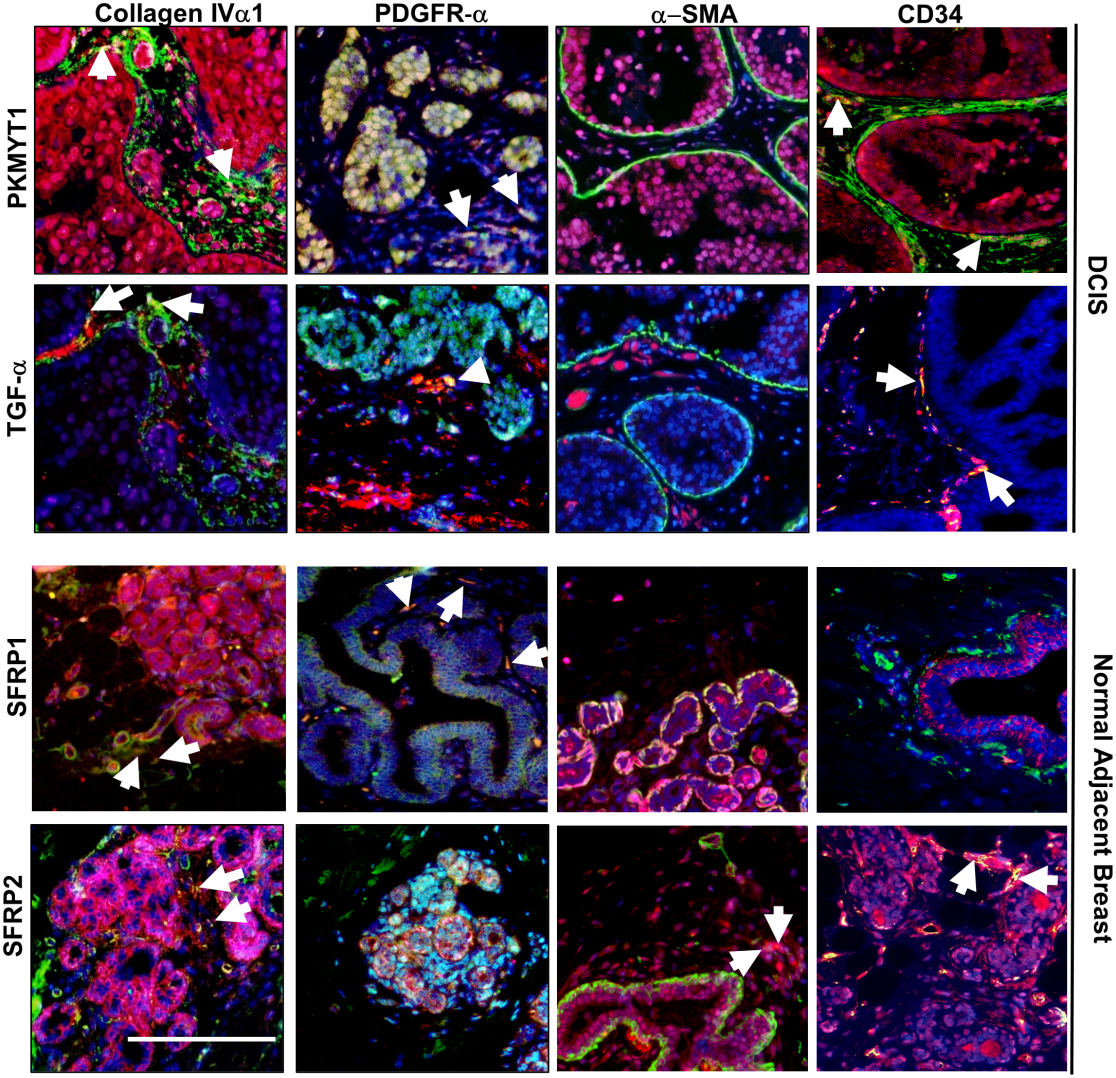
Co-immunofluorescence staining of PKMYT1, TGF-α, SFRP1 and SFRP2 with fibroblast associated biomarkers. DCIS or normal adjacent breast tissue were co-stained with PKMYT1, TGF-α, SFRP1 or SFRP2 (red) with the indicated fibroblast markers (green). Sections were counterstained with DAPI. White arrows indicate overlapping expression (yellow). Scale bar = 200 microns.

Finally, we sought to further characterize which fibroblast subtypes might be associated with PKMYT1, TGF-α, SFRP1 and SFRP2 expression through co-immunofluorescence staining of breast tissues. Previous studies have reported inverse associations between CD34 expression in DCIS and IDC stroma. With DCIS, higher α-SMA and lower CD34 expression in the stroma were more prevalent in intermediate and high-grade cases. In IDC, elevated α-SMA and loss of CD34 expression in fibroblastic stroma was observed (9, 16, 38, 39). Here, we characterized their expression in normal breast, DCIS, and IDC stroma. Notably, α−SMA expression was detected in the ductal myoepithelium of normal breast and DCIS tissues and in the periductal stroma of DCIS and IDC tissues. CD34 expression was localized to periductal stromal cells, such as endothelial or fibroblastic cells of normal breast, DCIS, and IDC tissues. In normal breast tissues, stromal expression of α-SMA appeared to be primarily absent while stromal expression of CD34 was strongly detected. With DCIS, higher α-SMA expression and lower CD34 expression in the stroma was associated with increased grade of disease. In IDC, increased α-SMA and loss of CD34 in fibroblastic stroma was observed, although CD34 expression appeared to be retained in the vasculature (**Supplemental Figure 8**). These observations were consistent with previously reported studies. We then co-stained for PKMYT1, TGF-α, SFRP1 and SFRP2 with the fibroblast markers: Collagen IVα1, PDGFR-α and α-SMA, which were respectively associated with ECM remodeling, immune modulation/M2 polarization and tumor proliferation/immune suppression (12–14). We also determined an association between expression of these gene targets with stromal expression of CD34. PKMYT1 and TGF-α expression partially overlapped with CollagenIVα1, PDGFR-α and CD34 but not with α-SMA. SFRP1 expression partly co-localized with Collagen IVα1, PDGFR-α but not with α-SMA or CD34 expressing cells. SFRP2 expression partially overlapped with ColIagenIVα1, α-SMA and CD34 but not with PDGFR-α (**Figure 6**). In summary, we observed partial overlap of the gene targets with more than one fibroblast biomarker.

### DCIS fibroblasts and IDC fibroblasts exert different effects on breast cancer progression

**Figure 7:**
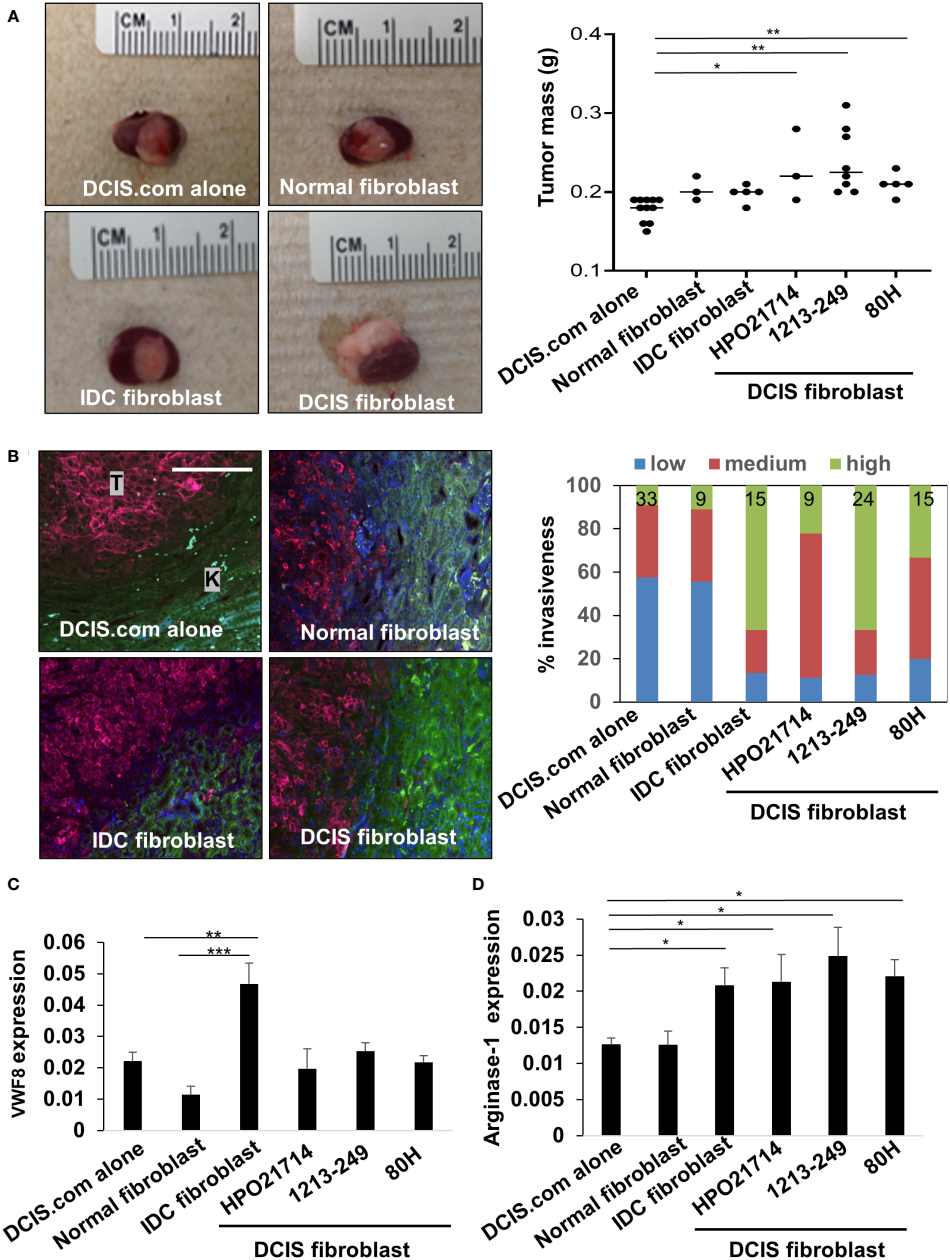
Effect of breast fibroblasts on DCIS.com breast cancer progression and stromal reactivity. DCIS.com cells were grafted with/without fibroblasts in the subrenal capsule of NOD SCID mice from normal breast (patient no. 2525), IDC (patient no.2760) or DCIS tissues for up to 21 days. Tumor tissues were harvested and analyzed for the following. **(A)** Tumor mass-representative images are shown including co-transplantation with 2525 normal fibroblasts, 2760 IDC fibroblasts, and 1213-249 DCIS fibroblasts. **(B)** Invasion was scored in tissues co-stained with phalloidin (green) and pan-cytokeratin (far red). Tumor tissue is indicated by “T.” Kidney is indicated by “K.” Percent invasiveness of grafts was determined by the total number of sections scored per sample. N sections/sample is indicated at the top of each bar in the graph. **(C, D)**. Tissues were immunostained for expression of Von Willebrand Factor 8 (VWF8) **(C)** and arginase 1 **(D)**. Expression was quantified by Image J (arbitrary units). Statistical analysis was performed using Kruskal Wallis test with Dunn’s *post-hoc* comparison **(A, C, D)** Statistical significance was determined by p-value <0.05. *p-value<0.05, **p-value<0.01, ***p-value<0.001. Scale bar = 20 microns. Mean ± SEM are shown.

To determine the functional contribution of DCIS fibroblasts to breast cancer progression, fibroblasts from individual cases of normal breast, DCIS or IDC tissues were co-grafted with DCIS.com breast cancer cells in NOD SCID mice, using the kidney capsule model. The kidney capsule is devoid of fibroblasts enabling reliable examination of transplanted cells without interference from host endogenous cells (30, 40). N=3-8 mice were grafted per case depending on the number of fibroblasts available after passaging and RNA sequencing. To control for the effects of fibroblasts, DCIS.com cells were grafted alone (n=11). After 17 days, tumors were evaluated for changes in growth and invasiveness. DCIS fibroblasts, normal breast, and IDC fibroblasts significantly enhanced DCIS.com tumor growth compared to tumor cells grafted alone (**Figure 7A**). To analyze for tumor invasion, tissues were co-stained for phalloidin and pan-cytokeratin, which were previously shown to distinguish breast epithelial cells from kidney cells (19). Tissues were scored based on definition of the tumor border and extent of tumor cell invasion into kidney parenchyma. DCIS fibroblasts enhanced tumor invasion compared to DCIS.com cells grafted alone or co-grafted with normal fibroblasts. IDC fibroblasts enhanced tumor invasion the most significantly (**Figure 7B**). In summary, these data indicate that DCIS fibroblasts enhance tumor growth similarly to both normal fibroblasts and IDC fibroblasts while enhancing tumor invasion over normal breast fibroblasts.

To determine how fibroblasts affected the microenvironment, we analyzed for changes in tumor angiogenesis and changes in arginase-1+ myeloid cells, cells that are typically associated with a pro-tumor response (41). DCIS fibroblasts significantly enhanced recruitment of arginase-1+ cells but did not significantly affect tumor angiogenesis as indicated by VWF8 expression, compared to DCIS.com cells alone and normal breast fibroblasts (**Figures 7C, D**). IDC fibroblasts significantly enhanced tumor angiogenesis and increased the levels of arginase-1+ cells compared to DCIS fibroblasts, normal fibroblasts and DCIS.com tumor cells grafted alone. Normal breast fibroblasts did not significantly affect tumor angiogenesis or recruitment of arginase-1+ cells. In summary, DCIS fibroblasts and IDC fibroblasts enhance recruitment of arginase-1+ cells and exert different effects on tumor angiogenesis.

## Discussion

To date, there are no reliable approaches to determine which cases of DCIS are more likely to become invasive. While fibroblasts are a major cellular component of the breast tumor microenvironment, their significance to DCIS progression has remained poorly understood. This study characterized the molecular profiles of normal, DCIS and IDC fibroblasts, and elucidate the functional contributions of DCIS fibroblasts to breast cancer progression. Through transcriptome profiling, we found that DCIS fibroblasts were characterized by unique alterations in cell cycle and motility related genes in such as PKMYT1, TGF-α, SFRP1 and SFRP2, which predicted increased cell growth and invasion by IPA. Immunostaining analysis revealed corresponding increases in expression of stromal derived PKMYT1, TGF-α and corresponding decreases expression of SFRP1 and SFRP2 in DCIS and IDC tissues. Through xenograft models, we demonstrated that co-transplantation with DCIS fibroblasts enhanced breast cancer growth and invasion associated with increased recruitment of arginase-1+ cells. In summary, these studies provide functional insight into the role of DCIS fibroblasts in breast cancer progression and identify some key biomarkers associated with DCIS progression to IDC, with important clinical implications.

One goal of this study was to determine whether DCIS fibroblasts were functionally similar or different from normal or IDC fibroblasts. The xenograft experiments showed that normal, DCIS and IDC fibroblasts enhanced the growth of DCIS.com xenografts compared to DCIS.com cells grafted alone, indicating some similarities in function among fibroblasts. However, we also observed many distinguishing characteristics of DCIS fibroblasts. For one, the *in vivo* experiments revealed that DCIS fibroblasts enhanced breast cancer invasion over normal fibroblasts, but less than IDC Fibroblasts. DCIS fibroblast-mediated tumor growth and invasion were associated with increased recruitment of arginase-1+ cells. In contrast, IDC fibroblast-mediated tumor growth and invasion was associated with both tumor angiogenesis and recruitment of arginase-1+ cells. In addition, RNA seq analysis revealed significant differences in gene expression between DCIS fibroblasts vs. normal fibroblasts and DCIS fibroblasts vs. IDC fibroblasts. At the protein level, we also detected differences in PKMYT1, TGF-α, SFRP1 and SFRP1 expression in normal, DCIS and IDC stroma. Overall, these data suggest that DCIS fibroblasts are different from normal breast and IDC fibroblasts.

Previous studies have indicated that ER expression in DCIS is associated with disease recurrence (7, 8). Here, we found that DCIS fibroblasts enhanced DCIS.com growth and invasion *in vivo*. Therefore, we considered whether ER status was associated with gene expression in DCIS fibroblasts, and whether ER status in DCIS might be associated with the tumor promoting effects of fibroblasts. By PCA and hierarchical clustering analysis of the heatmap, fibroblasts did not segregate exclusively by ER status. Through the *in vivo* experiments, fibroblasts from ER- DCIS (80H) and ER+ DCIS (1213249) enhanced DCIS.com growth and invasion to similar levels. At this time, we cannot conclude that ER expression alone is a contributing factor to the DCIS fibroblast phenotypes. To determine this possibility, studies would need to be performed on fibroblasts from a larger sample of ER+ and ER- DCIS cases.

While transcriptomics analysis revealed differences among types of fibroblasts, we also observed a certain level of variation in gene expression within groups including normal fibroblasts and IDC fibroblasts. The variation in normal fibroblasts is consistent with previously reported observations of gene expression in breast stroma (14, 33). Differences within normal fibroblasts could also be due to origin of tissues, as some normal fibroblasts were isolated from tissues adjacent to carcinoma and from normal breast tissues obtained from reduction mammoplasty. For IDC fibroblasts, we considered molecular subtype and disease stage as potential factors for their heterogeneity. The effects of molecular subtype in gene expression have previously been documented (7, 42–44). By PCA, fibroblasts that originated from triple negative IDC, such as 8661 and 2760 grouped more closely together than fibroblasts 8870 and 8727, which originated from ER+/PR/HER2+ IDC tissues. However, hierarchical clustering analysis still revealed differences in gene expression between fibroblasts from the same breast cancer subtypes. By contrast, we found that fibroblasts from the same stage of IDC, such as 2300 and 8727 were dissimilar. These data indicate that molecular subtype may be a stronger influencing factor than stage, but it is not the only factor influencing stromal gene expression. Biological factors such as genetics, age and breast density (45–47) and lifestyle factors such as diet (48) could potentially affect gene expression and contribute to heterogeneity of fibroblasts within groups. These same factors as well as technical factors in isolation and culture of fibroblasts and platforms used could have contributed to overall differences between our experimental dataset and published datasets. Despite the variations seen, we were still able to identify statistically significant differences in gene networks among fibroblast groups and validate several gene targets by immunostaining.

Here, we show that DCIS fibroblasts show increased expression of cell growth, cell cycle related genes such as TGF-α and PKMYT1. The role of TGF-α in late-stage breast cancer is well documented. As a member of the Epidermal Growth Factor family of ligands, TGF-α binds to ErbB1 receptor tyrosine kinases to promote breast tumor growth and progression (49–51). As an extracellular factor expressed by fibroblasts in DCIS, TGF-α could act on breast cancer cells to promote the growth and invasion of DCIS lesions. How might stromal expression of intracellular genes such as PKMYT1 function in disease progression? PKMYT1 is a cell checkpoint protein, which acts by phosphorylating and inhibiting CDK1 activity. This enables DNA repair and prevents pre-mature entry into mitosis and subsequent cell death (52, 53). In tumor cells, by arresting the cycle, PKMYT1 expression is important for cell survival and growth (54–56). In DCIS, tissues can undergo environmental stresses such as hypoxia, acidity, and low nutrient levels (57). As such, we propose that in DCIS, fibroblasts may express PKMYT1 to mediate cell cycle arrest, and support cell replication and survival during these stressful conditions. This would enable fibroblasts to support a pro-cancerous niche through secretion of growth factors like TGF-α. Further studies would need to be performed to characterize the function of cell cycle checkpoint pathways in breast fibroblasts during DCIS progression.

Our studies demonstrated a decrease in stromal SFRP1 and SFRP2 expression in breast cancer stroma, with the lowest levels observed in IDC tissues. The decreased expression is consistent with previous studies showing downregulation of overall SFRP1 and SFRP2 expression in breast tumors through DNA promoter hypermethylation (58, 59). Interestingly, we found that stromal SFRP2 was negatively associated with PR and HER2 expression in DCIS but not IDC, suggesting an association with subtype in early-stage disease. What is the potential significance of stromal SFRP1 and SFRP2 expression in breast cancer? As secreted glycoproteins, SFRP1 and SFRP2 are thought to be primarily tumor suppressive as they bind and sequester WNT ligands to modulate cancer cell growth, cell polarity, transformation and cancer stem cell activity (60). However, tumor promoting roles for SFRP1 and SFRP2 have been reported in cancer. Methylation of SFRP1 is associated with poor prognosis in ER+/HER2+ breast cancers (61). Overexpression of SFRP1 in gastric cancer cells enhance cell growth and migration through Rac and GSK3β dependent mechanisms (62). SFRP2 antibody neutralization inhibits the growth of triple negative breast tumor xenografts associated with activation of beta catenin (63, 64). SFRP2 enhances WNT16 signaling to promote prostate growth and therapeutic resistance (65). In our studies, stromal SFRP2 correlated with Ki67 and stromal CCL2 expression in DCIS. In IDC, lower stromal SFRP1 expression was associated with survival. These data suggest a cancer promoting role for stromal SFRP1 and 2 protein expression in DCIS and IDC.

How might stromal SFRP1 and SFRP2 facilitate breast cancer progression? Previous studies have shown that SFRP can enhance or inhibit WNT signaling depending on concentration (66–68). In addition to their extracellular roles, SFRP also functions in the nucleus by binding to beta catenin at different domains to inhibit or promote activity (69). As such, the reduced levels of stromal SFRP1 and SFRP2 in DCIS and IDC may facilitate WNT signaling and promote breast cancer growth, survival, invasion, or stem cell activity. SFRPs may also enhance tumor angiogenesis through non-canonical WNT signaling (59) and regulating macrophage recruitment during inflammation (70). As such, SFRP1 and SFRP2 expression in fibroblasts could serve to regulate breast cancer progression by acting on epithelial cells and other cells in the tumor microenvironment. More functional studies on stromal SFRP1 and SFRP2 should be performed to further clarify their roles in stage specific disease progression.

Our studies revealed similarities and differences between RNA and protein expression of stromal biomarkers across the different breast tissue groups. RNA and protein levels were similar with elevated PKMYT1 and TGF-α in DCIS fibroblasts and stromal tissues compared to normal fibroblasts and stromal tissues, and higher SFRP1 and SFRP2 expression in DCIS fibroblasts and stromal tissues compared to IDC fibroblasts and stroma. However, TGFA RNA levels were higher in DCIS fibroblasts vs. IDC fibroblasts while TGF-α protein levels were lower in DCIS stroma vs. IDC stroma in tissues. These discrepancies between RNA and protein expression of biomarkers are not unusual. Previous studies have demonstrated that differences between RNA and protein expression of biomarkers are common (71–73). This could be due to several factors. Protein stability of TGF-α protein could be affected by post-transcriptional mechanisms such as microRNAs (74, 75) and post-translational modifications through glycosylation and palmitoylation (76, 77). In addition, conditions present in whole tissues such as stromal: tumor interactions could have influenced gene expression of epidermal growth factors (78–80). Furthermore, TGF-α could be expressed in myeloid cells in the microenvironment such as macrophages and neutrophils, contributing to differences in overall expression in tissue stroma (49, 81, 82). Altogether, these studies reveal insight into RNA and protein expression in breast stroma.

Overall, we demonstrate significant gene expression differences in DCIS fibroblasts associated with increased tumor growth, invasion, and stromal reactivity. The identification of biomarkers specific to DCIS and IDC stroma enhance our understanding of how the stroma evolves with disease progression and could have value identifying which cases of DCIS are at risk for becoming invasive. However, at this time, we cannot draw conclusions about their value as predictive markers to identify high-risk DCIS cases, due to the absence of follow-up outcomes on DCIS cases. To advance these stromal biomarkers as predictive biomarkers, future studies would need to be performed in a larger cohort of DCIS cases that include outcome data on disease recurrence, progression, and survival. Furthermore, it would be of interest to follow expression of the proposed biomarkers to DCIS progression in animal models. For example, if we examined sufficient cases of patient DCIS or animal models and found that high expression of TGF-α, PKMYT1 and decreased SFRP1 and SFRP2 in the stroma were associated with disease recurrence, invasive disease or decreased survival, these data could indicate predictive value for these markers. Success of these studies could lead to more effective approaches to identify high risk patients and tailor more effective and safer treatment regimens to prevent or treat IDC.

## Data availability statement

The RNA seq data discussed in this study have been deposited in NCBI’s Gene Expression Omnibus with the GEO Series accession number GSE228582. The url is https://www.ncbi.nlm.nih.gov/geo/query/acc.cgi?acc=GSE228582.

## Ethics statement

The studies involving human participants were reviewed and approved by KUMC Human Research Protection Program. The ethics committee waived the requirement of written informed consent for participation. The animal study was reviewed and approved by KUMC Institutional Animal Care and Use Committee.

## Author contributions

WF and NC were involved in study design, data analysis and interpretation. MP performed animal experiments. PC, MP, and MM performed immunostaining. WF cultured cells, isolated RNA and performed RT PCR analysis. YH and FB were involved in tissue collection. WF, YH, and FB were involved in fibroblast isolation. CB performed quality control tests for RNA and performed the RNA seq. JK processed transcriptomics data. WF, NC, JN, and VR reviewed data. WF, NC, JN-M, and VR wrote, assembled, and edited the manuscript. All authors reviewed the manuscript prior to submission. All authors contributed to the article and approved the submitted version.
