# Supplementary material for: Transcriptome analysis reveals differences in cell cycle, growth and migration related genes that distinguish fibroblasts derived from pre-invasive and invasive breast cancer
Source: Front Oncol. 2023 Apr 6;13:1130911. doi: 10.3389/fonc.2023.1130911 (PMC10118028; doi:10.3389/fonc.2023.1130911)
Supplement: Supplementary file 1 [file Presentation_1.pptx]

## Slide 1
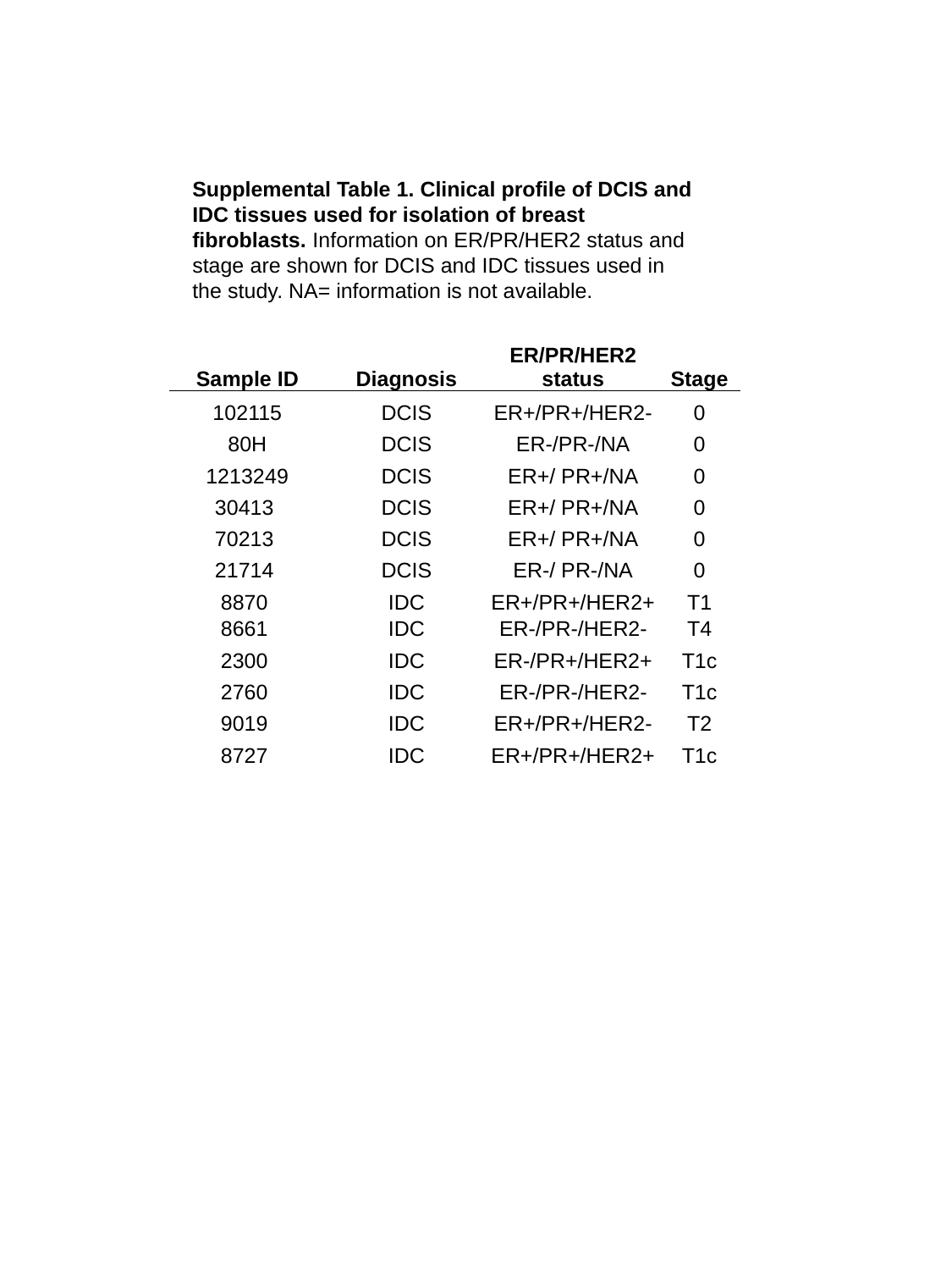

Supplemental Table 1. Clinical profile of DCIS and IDC tissues used for isolation of breast fibroblasts. Information on ER/PR/HER2 status and stage are shown for DCIS and IDC tissues used in the study. NA= information is not available.
| Sample ID | Diagnosis | ER/PR/HER2 status | Stage |
| --- | --- | --- | --- |
| 102115 | DCIS | ER+/PR+/HER2- | 0 |
| 80H | DCIS | ER-/PR-/NA | 0 |
| 1213249 | DCIS | ER+/ PR+/NA | 0 |
| 30413 | DCIS | ER+/ PR+/NA | 0 |
| 70213 | DCIS | ER+/ PR+/NA | 0 |
| 21714 | DCIS | ER-/ PR-/NA | 0 |
| 8870 | IDC | ER+/PR+/HER2+ | T1 |
| 8661 | IDC | ER-/PR-/HER2- | T4 |
| 2300 | IDC | ER-/PR+/HER2+ | T1c |
| 2760 | IDC | ER-/PR-/HER2- | T1c |
| 9019 | IDC | ER+/PR+/HER2- | T2 |
| 8727 | IDC | ER+/PR+/HER2+ | T1c |

## Slide 2
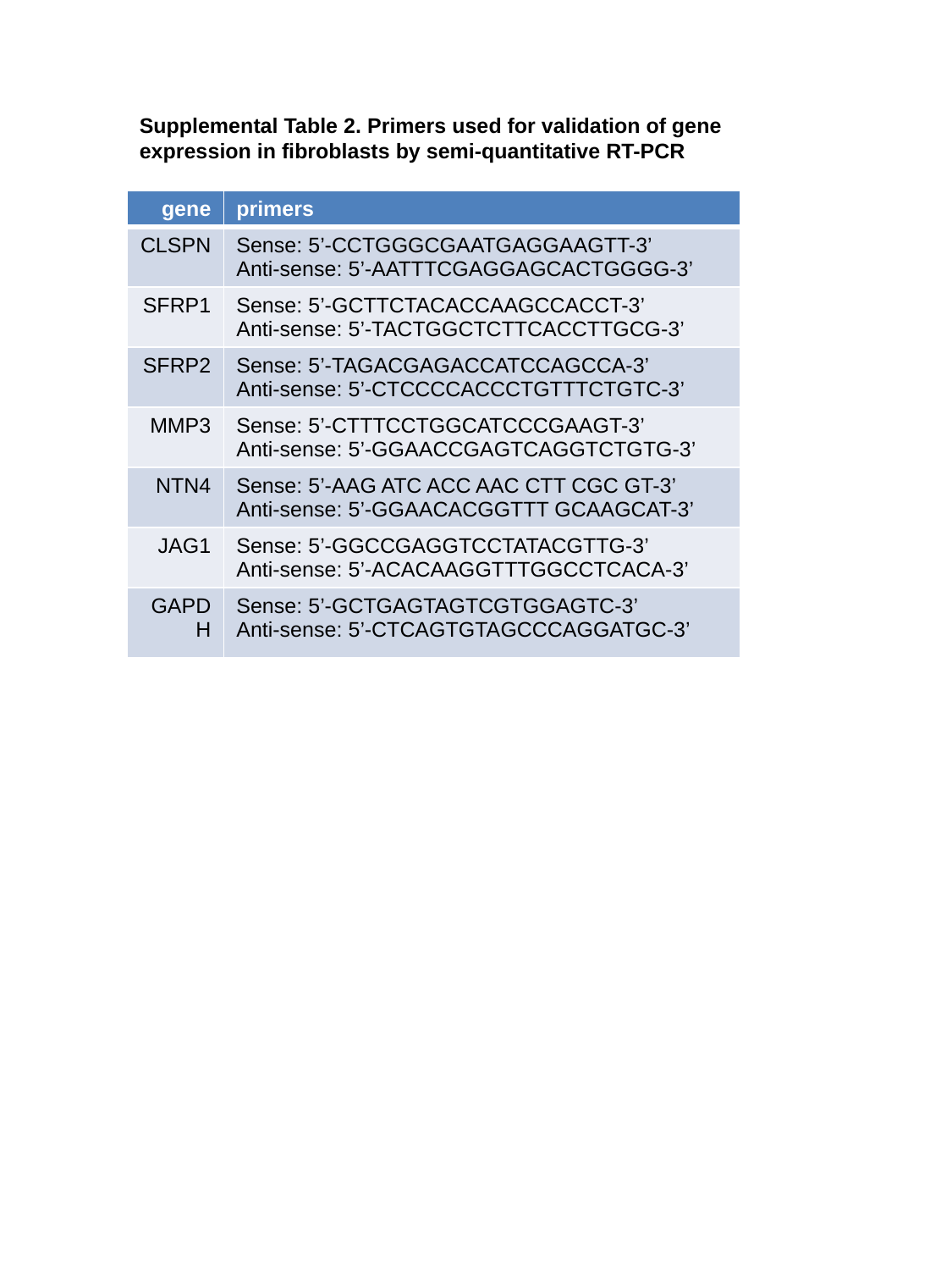

Supplemental Table 2. Primers used for validation of gene expression in fibroblasts by semi-quantitative RT-PCR
| gene | primers |
| --- | --- |
| CLSPN | Sense: 5’-CCTGGGCGAATGAGGAAGTT-3’ Anti-sense: 5’-AATTTCGAGGAGCACTGGGG-3’ |
| SFRP1 | Sense: 5’-GCTTCTACACCAAGCCACCT-3’ Anti-sense: 5’-TACTGGCTCTTCACCTTGCG-3’ |
| SFRP2 | Sense: 5’-TAGACGAGACCATCCAGCCA-3’ Anti-sense: 5’-CTCCCCACCCTGTTTCTGTC-3’ |
| MMP3 | Sense: 5’-CTTTCCTGGCATCCCGAAGT-3’ Anti-sense: 5’-GGAACCGAGTCAGGTCTGTG-3’ |
| NTN4 | Sense: 5’-AAG ATC ACC AAC CTT CGC GT-3’ Anti-sense: 5’-GGAACACGGTTT GCAAGCAT-3’ |
| JAG1 | Sense: 5’-GGCCGAGGTCCTATACGTTG-3’ Anti-sense: 5’-ACACAAGGTTTGGCCTCACA-3’ |
| GAPDH | Sense: 5’-GCTGAGTAGTCGTGGAGTC-3’ Anti-sense: 5’-CTCAGTGTAGCCCAGGATGC-3’ |

## Slide 3
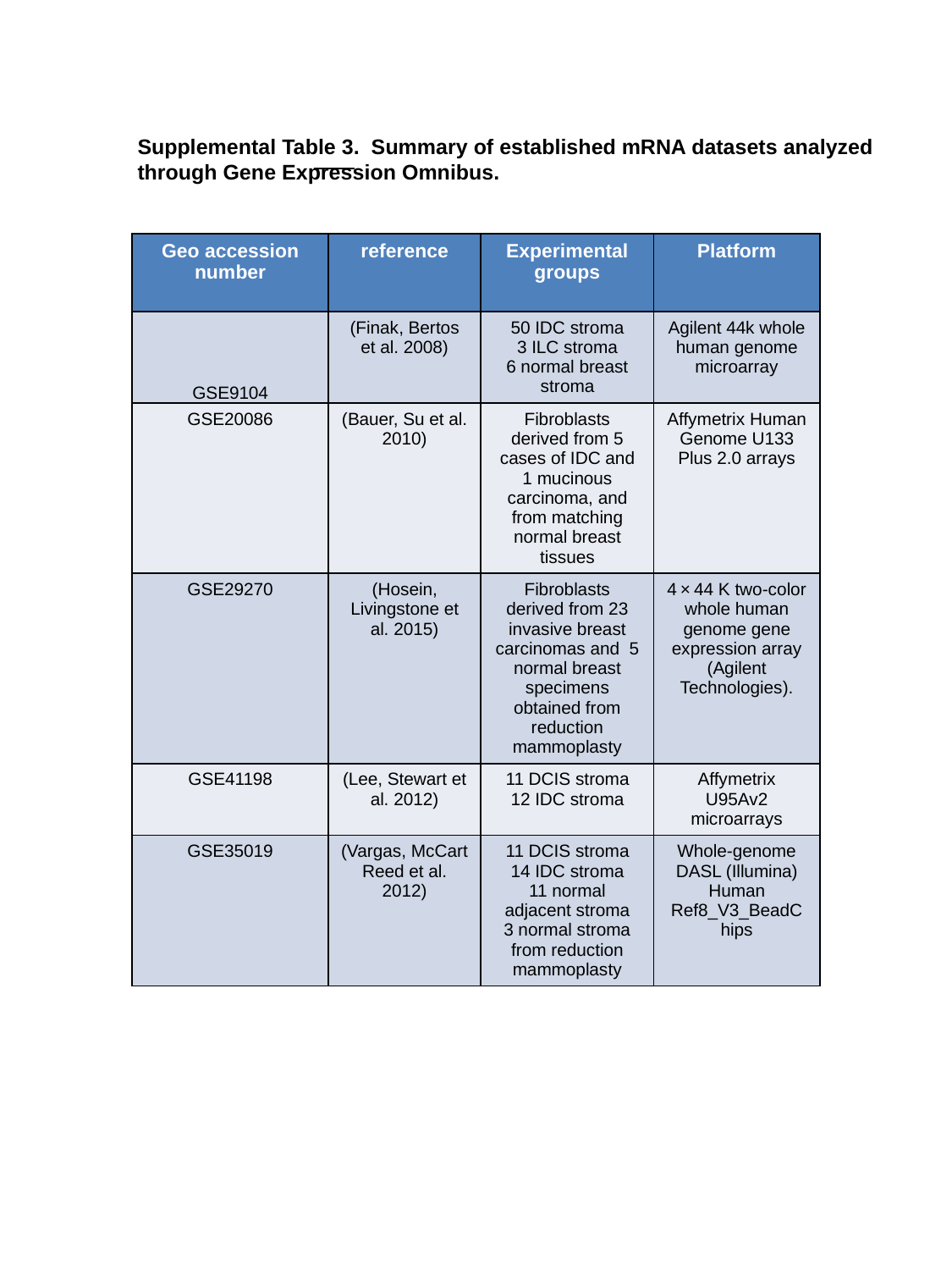

Supplemental Table 3. Summary of established mRNA datasets analyzed through Gene Expression Omnibus.
| Geo accession number | reference | Experimental groups | Platform |
| --- | --- | --- | --- |
| GSE9104 | (Finak, Bertos et al. 2008) | 50 IDC stroma 3 ILC stroma 6 normal breast stroma | Agilent 44k whole human genome microarray |
| GSE20086 | (Bauer, Su et al. 2010) | Fibroblasts derived from 5 cases of IDC and 1 mucinous carcinoma, and from matching normal breast tissues | Affymetrix Human Genome U133 Plus 2.0 arrays |
| GSE29270 | (Hosein, Livingstone et al. 2015) | Fibroblasts derived from 23 invasive breast carcinomas and 5 normal breast specimens obtained from reduction mammoplasty | 4 × 44 K two-color whole human genome gene expression array (Agilent Technologies). |
| GSE41198 | (Lee, Stewart et al. 2012) | 11 DCIS stroma 12 IDC stroma | Affymetrix U95Av2 microarrays |
| GSE35019 | (Vargas, McCart Reed et al. 2012) | 11 DCIS stroma 14 IDC stroma 11 normal adjacent stroma 3 normal stroma from reduction mammoplasty | Whole-genome DASL (Illumina) Human Ref8\_V3\_BeadChips |

## Slide 4
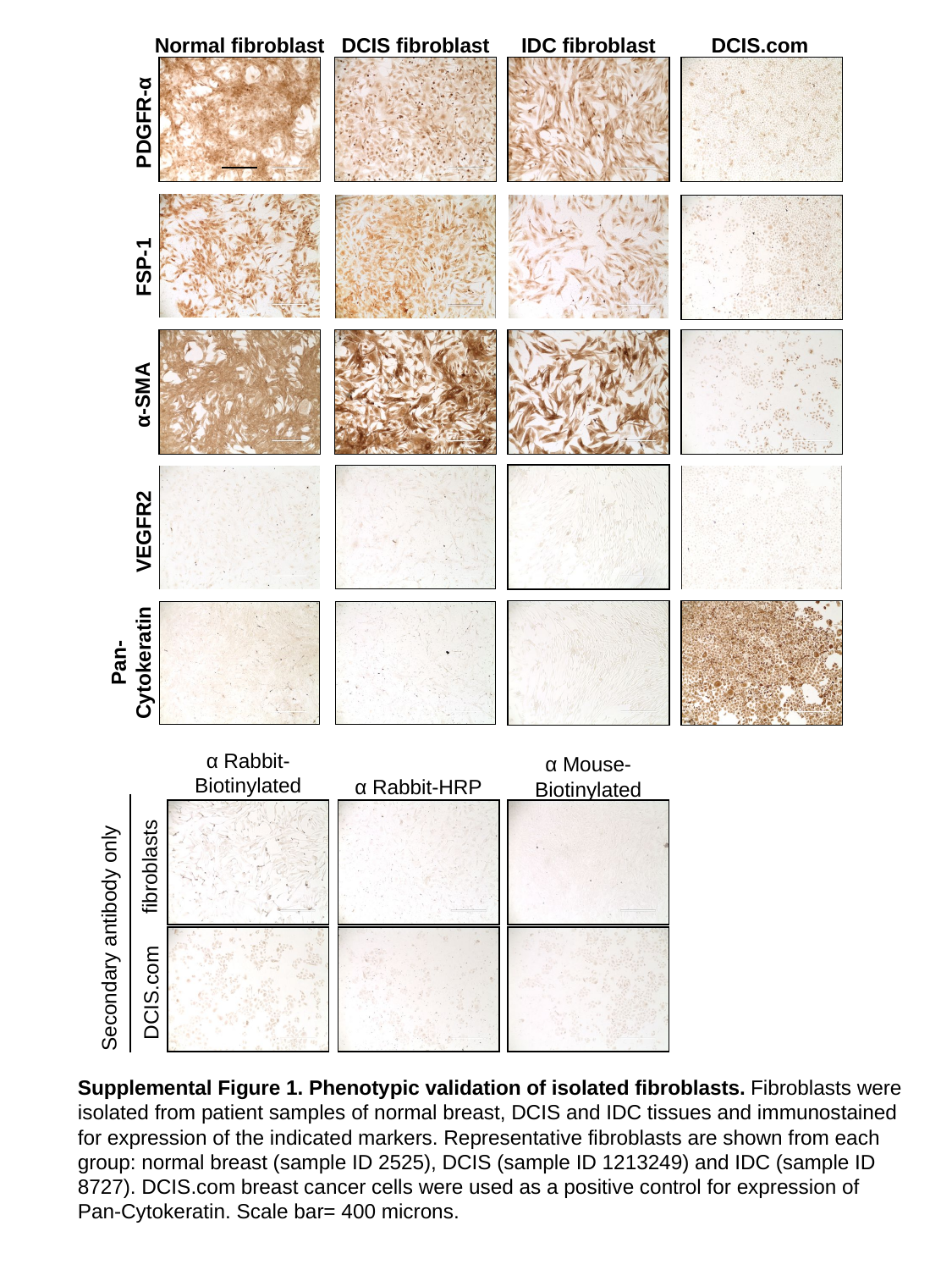

Normal fibroblast
DCIS fibroblast
IDC fibroblast
DCIS.com
PDGFR-α
FSP-1
α-SMA
VEGFR2
Pan-Cytokeratin
α Rabbit- Biotinylated
α Mouse- Biotinylated
α Rabbit-HRP
fibroblasts
DCIS.com
Secondary antibody only
Supplemental Figure 1. Phenotypic validation of isolated fibroblasts. Fibroblasts were isolated from patient samples of normal breast, DCIS and IDC tissues and immunostained for expression of the indicated markers. Representative fibroblasts are shown from each group: normal breast (sample ID 2525), DCIS (sample ID 1213249) and IDC (sample ID 8727). DCIS.com breast cancer cells were used as a positive control for expression of Pan-Cytokeratin. Scale bar= 400 microns.

## Slide 5
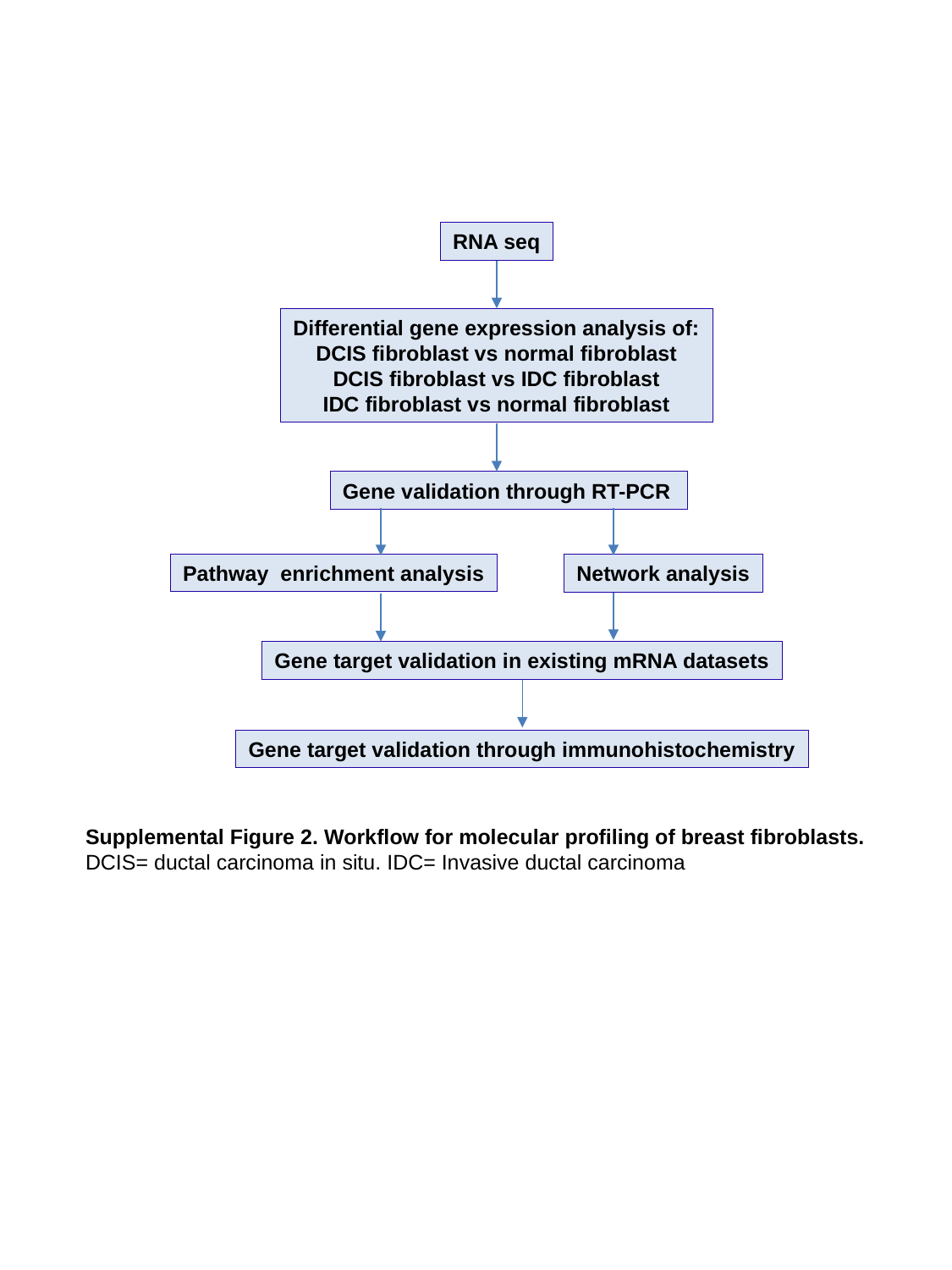

RNA seq
Differential gene expression analysis of:
DCIS fibroblast vs normal fibroblast
DCIS fibroblast vs IDC fibroblast
IDC fibroblast vs normal fibroblast
Gene validation through RT-PCR
Pathway enrichment analysis
Network analysis
Gene target validation in existing mRNA datasets
Gene target validation through immunohistochemistry
Supplemental Figure 2. Workflow for molecular profiling of breast fibroblasts.
DCIS= ductal carcinoma in situ. IDC= Invasive ductal carcinoma

## Slide 6
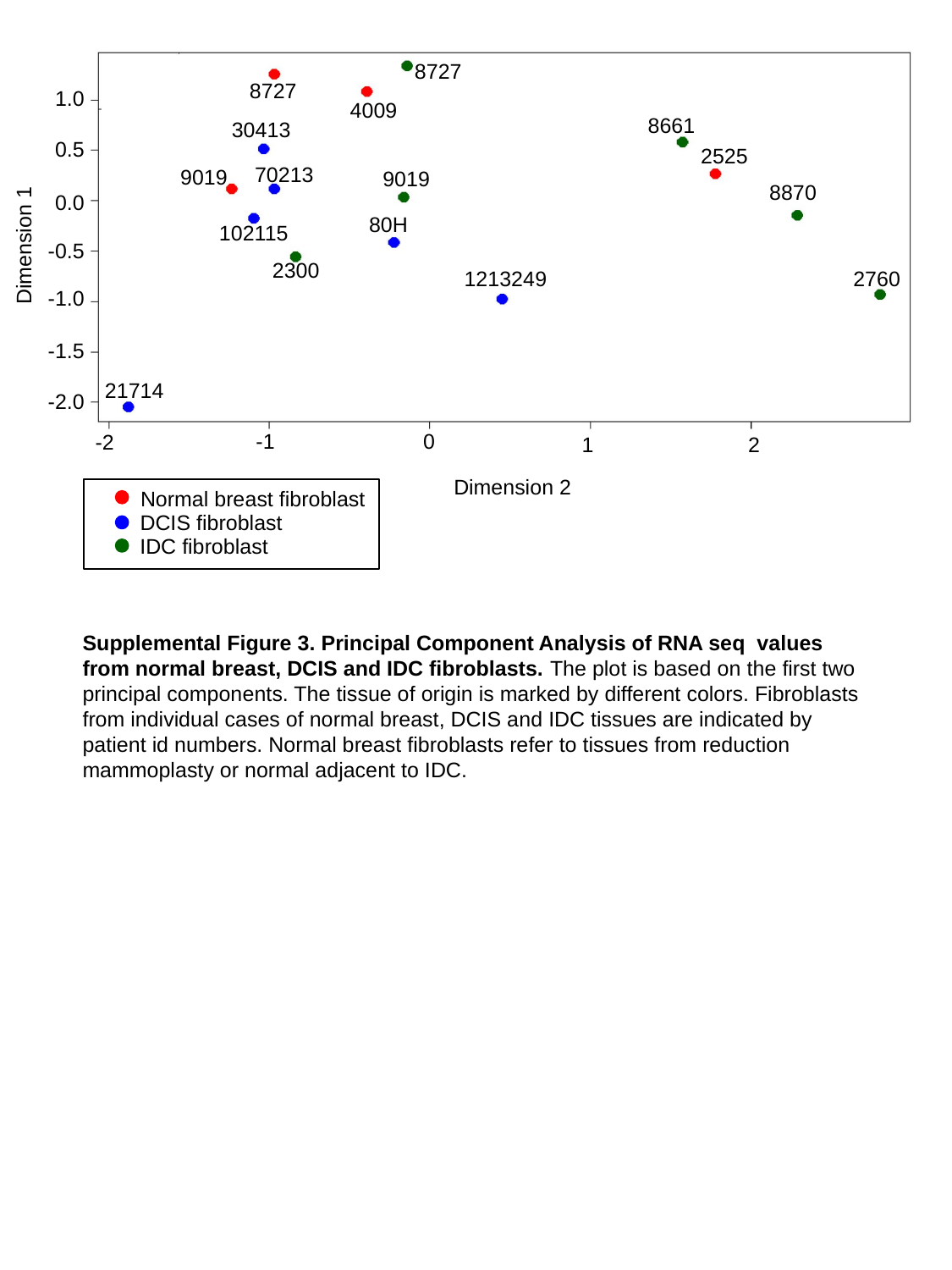

8727
8727
1.0
4009
8661
30413
0.5
2525
70213
9019
9019
8870
0.0
80H
102115
Dimension 1
-0.5
2300
2760
1213249
-1.0
-1.5
21714
-2.0
-1
0
-2
1
2
Dimension 2
Normal breast fibroblast
DCIS fibroblast
IDC fibroblast
Supplemental Figure 3. Principal Component Analysis of RNA seq values from normal breast, DCIS and IDC fibroblasts. The plot is based on the first two principal components. The tissue of origin is marked by different colors. Fibroblasts from individual cases of normal breast, DCIS and IDC tissues are indicated by patient id numbers. Normal breast fibroblasts refer to tissues from reduction mammoplasty or normal adjacent to IDC.

## Slide 7
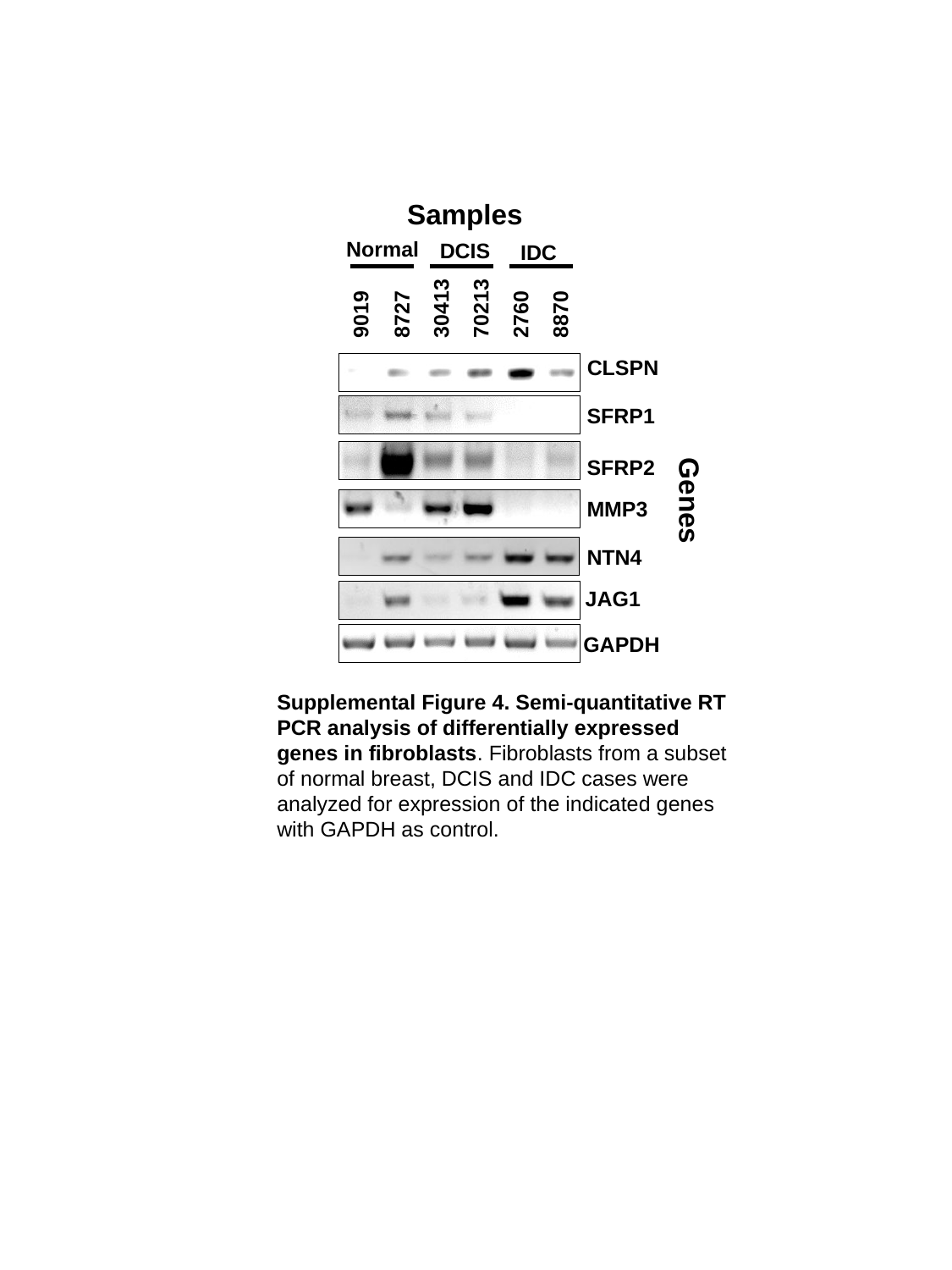

Samples
Normal
DCIS
IDC
30413
70213
9019
8727
2760
8870
CLSPN
SFRP1
SFRP2
Genes
MMP3
NTN4
JAG1
GAPDH
Supplemental Figure 4. Semi-quantitative RT PCR analysis of differentially expressed genes in fibroblasts. Fibroblasts from a subset of normal breast, DCIS and IDC cases were analyzed for expression of the indicated genes with GAPDH as control.

## Slide 8
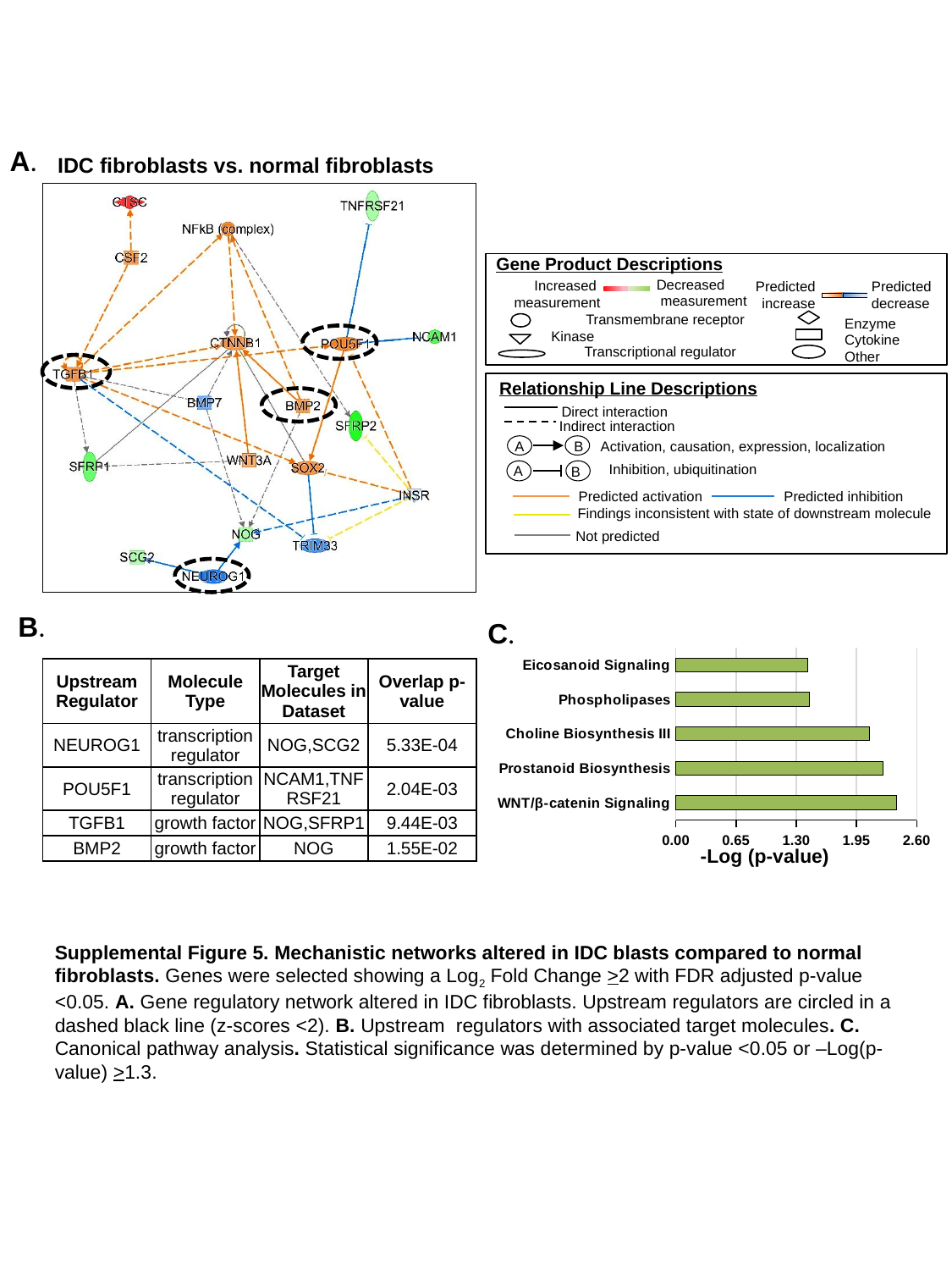

A.
IDC fibroblasts vs. normal fibroblasts
Gene Product Descriptions
Decreased
 measurement
Increased
measurement
Transmembrane receptor
Enzyme
Cytokine
Other
Kinase
Transcriptional regulator
Predicted
increase
Predicted
decrease
Relationship Line Descriptions
Direct interaction
Indirect interaction
B
A
Activation, causation, expression, localization
Inhibition, ubiquitination
A
B
Predicted inhibition
Predicted activation
Findings inconsistent with state of downstream molecule
Not predicted
B.
C.
### Chart
| Category | |
|---|---|
| WNT/β-catenin Signaling | 2.38 |
| Prostanoid Biosynthesis | 2.24 |
| Choline Biosynthesis III | 2.09 |
| Phospholipases | 1.44 |
| Eicosanoid Signaling | 1.42 |-Log (p-value)
| Upstream Regulator | Molecule Type | Target Molecules in Dataset | Overlap p-value |
| --- | --- | --- | --- |
| NEUROG1 | transcription regulator | NOG,SCG2 | 5.33E-04 |
| POU5F1 | transcription regulator | NCAM1,TNFRSF21 | 2.04E-03 |
| TGFB1 | growth factor | NOG,SFRP1 | 9.44E-03 |
| BMP2 | growth factor | NOG | 1.55E-02 |
Supplemental Figure 5. Mechanistic networks altered in IDC blasts compared to normal fibroblasts. Genes were selected showing a Log2 Fold Change >2 with FDR adjusted p-value <0.05. A. Gene regulatory network altered in IDC fibroblasts. Upstream regulators are circled in a dashed black line (z-scores <2). B. Upstream regulators with associated target molecules. C. Canonical pathway analysis. Statistical significance was determined by p-value <0.05 or –Log(p-value) >1.3.

## Slide 9
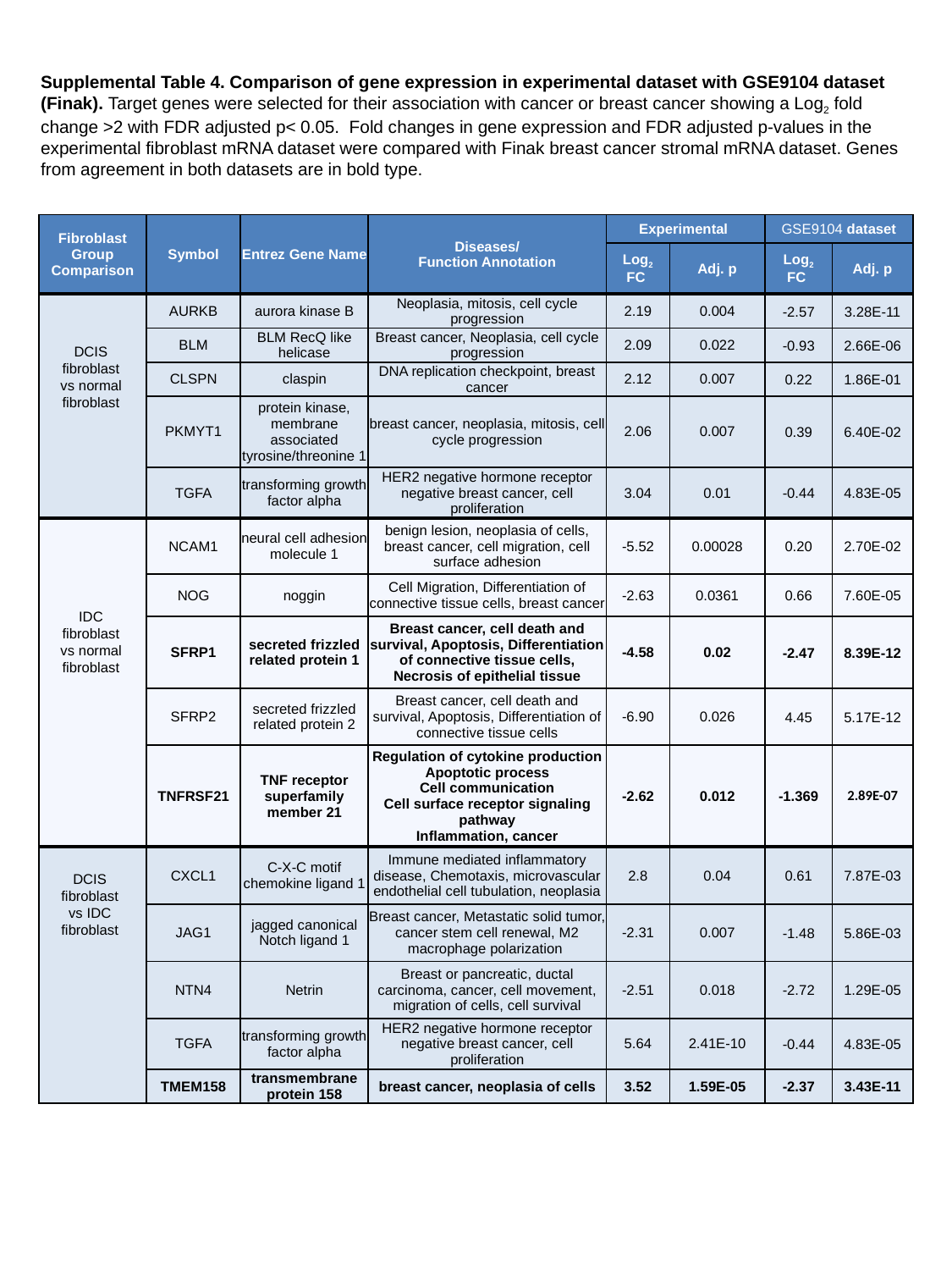

Supplemental Table 4. Comparison of gene expression in experimental dataset with GSE9104 dataset (Finak). Target genes were selected for their association with cancer or breast cancer showing a Log2 fold change >2 with FDR adjusted p< 0.05. Fold changes in gene expression and FDR adjusted p-values in the experimental fibroblast mRNA dataset were compared with Finak breast cancer stromal mRNA dataset. Genes from agreement in both datasets are in bold type.
| Fibroblast Group Comparison | Symbol | Entrez Gene Name | Diseases/ Function Annotation | Experimental | | GSE9104 dataset | |
| --- | --- | --- | --- | --- | --- | --- | --- |
| | | | | Log2 FC | Adj. p | Log2 FC | Adj. p |
| | AURKB | aurora kinase B | Neoplasia, mitosis, cell cycle progression | 2.19 | 0.004 | -2.57 | 3.28E-11 |
| | BLM | BLM RecQ like helicase | Breast cancer, Neoplasia, cell cycle progression | 2.09 | 0.022 | -0.93 | 2.66E-06 |
| | CLSPN | claspin | DNA replication checkpoint, breast cancer | 2.12 | 0.007 | 0.22 | 1.86E-01 |
| | PKMYT1 | protein kinase, membrane associated tyrosine/threonine 1 | breast cancer, neoplasia, mitosis, cell cycle progression | 2.06 | 0.007 | 0.39 | 6.40E-02 |
| | TGFA | transforming growth factor alpha | HER2 negative hormone receptor negative breast cancer, cell proliferation | 3.04 | 0.01 | -0.44 | 4.83E-05 |
| | NCAM1 | neural cell adhesion molecule 1 | benign lesion, neoplasia of cells, breast cancer, cell migration, cell surface adhesion | -5.52 | 0.00028 | 0.20 | 2.70E-02 |
| | NOG | noggin | Cell Migration, Differentiation of connective tissue cells, breast cancer | -2.63 | 0.0361 | 0.66 | 7.60E-05 |
| | SFRP1 | secreted frizzled related protein 1 | Breast cancer, cell death and survival, Apoptosis, Differentiation of connective tissue cells, Necrosis of epithelial tissue | -4.58 | 0.02 | -2.47 | 8.39E-12 |
| | SFRP2 | secreted frizzled related protein 2 | Breast cancer, cell death and survival, Apoptosis, Differentiation of connective tissue cells | -6.90 | 0.026 | 4.45 | 5.17E-12 |
| | TNFRSF21 | TNF receptor superfamily member 21 | Regulation of cytokine production Apoptotic process Cell communication Cell surface receptor signaling pathway Inflammation, cancer | -2.62 | 0.012 | -1.369 | 2.89E-07 |
| | CXCL1 | C-X-C motif chemokine ligand 1 | Immune mediated inflammatory disease, Chemotaxis, microvascular endothelial cell tubulation, neoplasia | 2.8 | 0.04 | 0.61 | 7.87E-03 |
| | JAG1 | jagged canonical Notch ligand 1 | Breast cancer, Metastatic solid tumor, cancer stem cell renewal, M2 macrophage polarization | -2.31 | 0.007 | -1.48 | 5.86E-03 |
| | NTN4 | Netrin | Breast or pancreatic, ductal carcinoma, cancer, cell movement, migration of cells, cell survival | -2.51 | 0.018 | -2.72 | 1.29E-05 |
| | TGFA | transforming growth factor alpha | HER2 negative hormone receptor negative breast cancer, cell proliferation | 5.64 | 2.41E-10 | -0.44 | 4.83E-05 |
| | TMEM158 | transmembrane protein 158 | breast cancer, neoplasia of cells | 3.52 | 1.59E-05 | -2.37 | 3.43E-11 |
DCIS fibroblast vs normal fibroblast
IDC fibroblast vs normal fibroblast
DCIS fibroblast vs IDC
fibroblast

## Slide 10
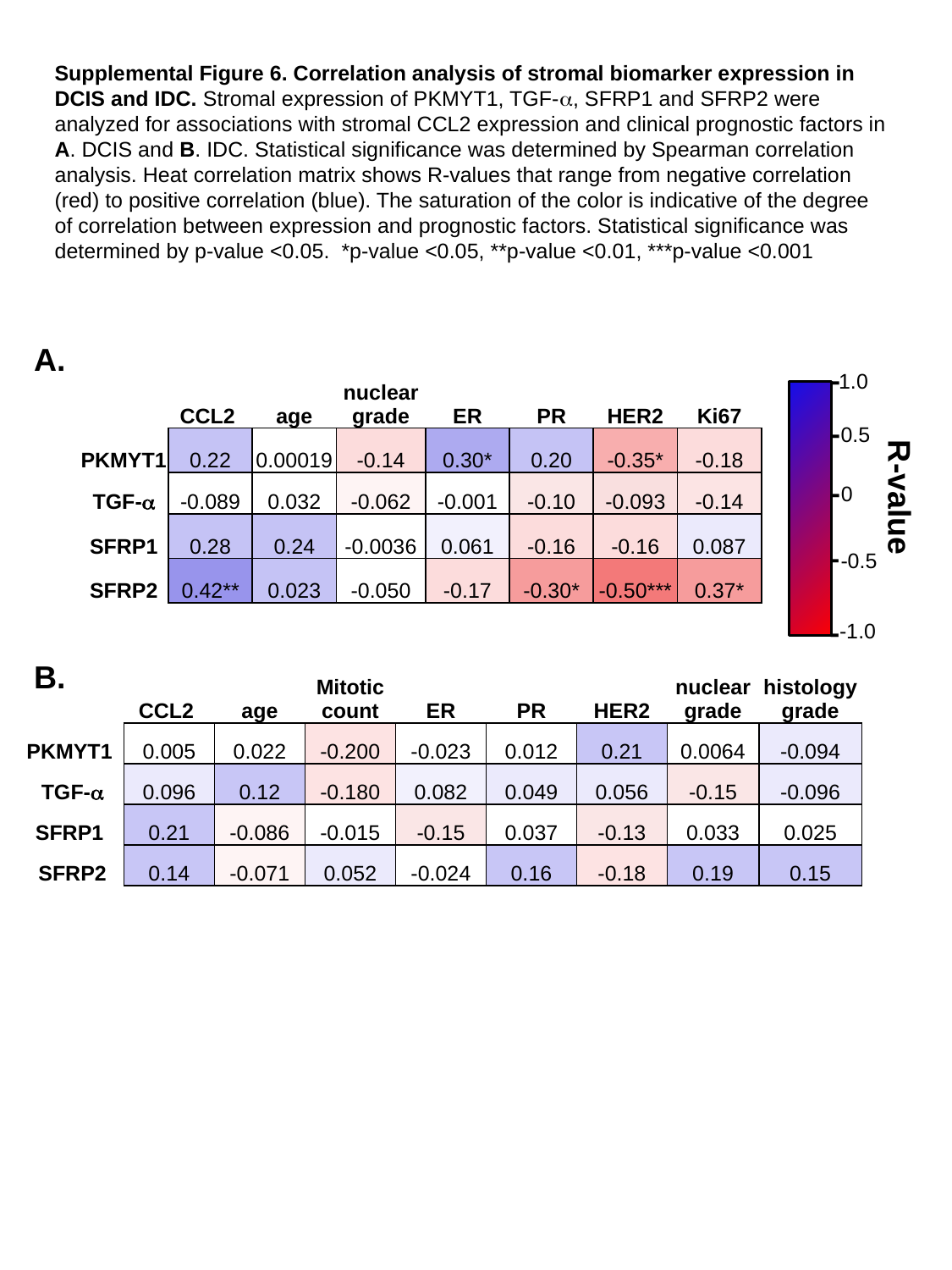

Supplemental Figure 6. Correlation analysis of stromal biomarker expression in DCIS and IDC. Stromal expression of PKMYT1, TGF-a, SFRP1 and SFRP2 were analyzed for associations with stromal CCL2 expression and clinical prognostic factors in A. DCIS and B. IDC. Statistical significance was determined by Spearman correlation analysis. Heat correlation matrix shows R-values that range from negative correlation (red) to positive correlation (blue). The saturation of the color is indicative of the degree of correlation between expression and prognostic factors. Statistical significance was determined by p-value <0.05. *p-value <0.05, **p-value <0.01, ***p-value <0.001
A.
-
1.0
-
0.5
-
0
-
-0.5
-
-1.0
| | CCL2 | age | nuclear grade | ER | PR | HER2 | Ki67 |
| --- | --- | --- | --- | --- | --- | --- | --- |
| PKMYT1 | 0.22 | 0.00019 | -0.14 | 0.30\* | 0.20 | -0.35\* | -0.18 |
| TGF-a | -0.089 | 0.032 | -0.062 | -0.001 | -0.10 | -0.093 | -0.14 |
| SFRP1 | 0.28 | 0.24 | -0.0036 | 0.061 | -0.16 | -0.16 | 0.087 |
| SFRP2 | 0.42\*\* | 0.023 | -0.050 | -0.17 | -0.30\* | -0.50\*\*\* | 0.37\* |
R-value
B.
| | CCL2 | age | Mitotic count | ER | PR | HER2 | nuclear grade | histology grade |
| --- | --- | --- | --- | --- | --- | --- | --- | --- |
| PKMYT1 | 0.005 | 0.022 | -0.200 | -0.023 | 0.012 | 0.21 | 0.0064 | -0.094 |
| TGF-a | 0.096 | 0.12 | -0.180 | 0.082 | 0.049 | 0.056 | -0.15 | -0.096 |
| SFRP1 | 0.21 | -0.086 | -0.015 | -0.15 | 0.037 | -0.13 | 0.033 | 0.025 |
| SFRP2 | 0.14 | -0.071 | 0.052 | -0.024 | 0.16 | -0.18 | 0.19 | 0.15 |

## Slide 11
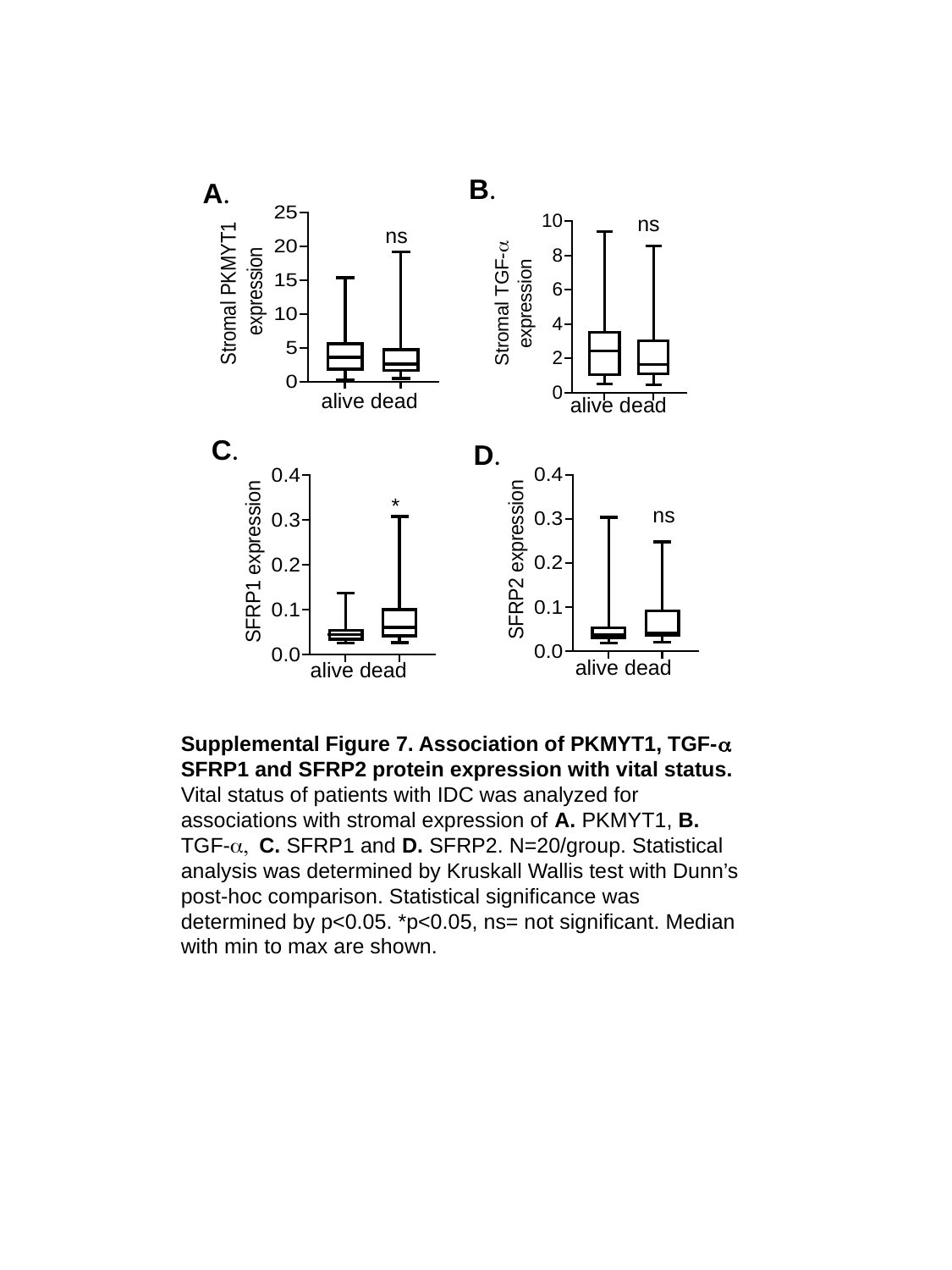

B.
A.
alive dead
ns
alive dead
ns
C.
D.
*
alive dead
alive dead
ns
Supplemental Figure 7. Association of PKMYT1, TGF-a SFRP1 and SFRP2 protein expression with vital status. Vital status of patients with IDC was analyzed for associations with stromal expression of A. PKMYT1, B. TGF-a, C. SFRP1 and D. SFRP2. N=20/group. Statistical analysis was determined by Kruskall Wallis test with Dunn’s post-hoc comparison. Statistical significance was determined by p<0.05. *p<0.05, ns= not significant. Median with min to max are shown.

## Slide 12
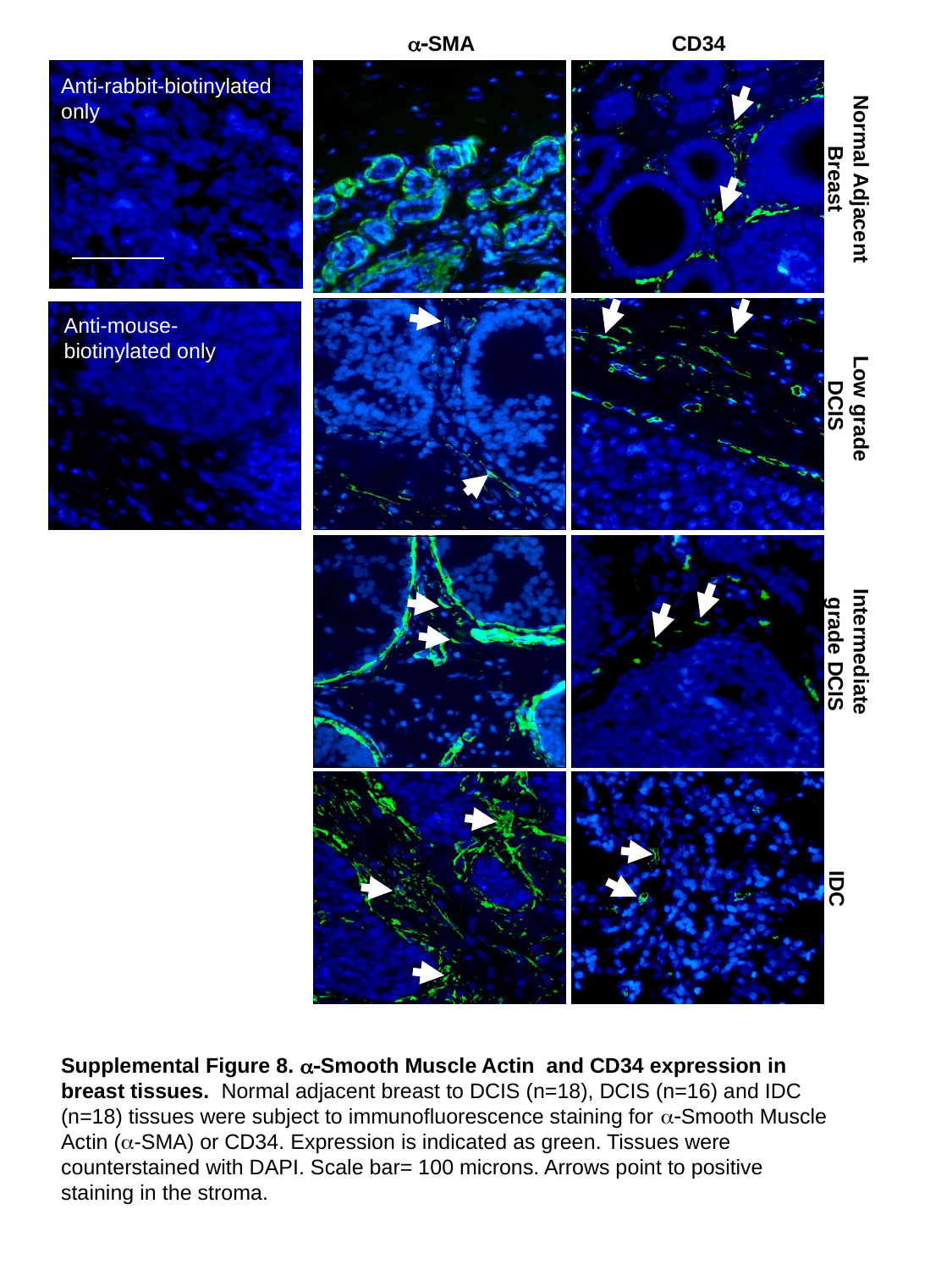

a-SMA
CD34
Anti-rabbit-biotinylated only
Normal Adjacent
Breast
Anti-mouse-biotinylated only
 Low grade DCIS
Intermediate grade DCIS
IDC
Supplemental Figure 8. a-Smooth Muscle Actin and CD34 expression in breast tissues. Normal adjacent breast to DCIS (n=18), DCIS (n=16) and IDC (n=18) tissues were subject to immunofluorescence staining for a-Smooth Muscle Actin (a-SMA) or CD34. Expression is indicated as green. Tissues were counterstained with DAPI. Scale bar= 100 microns. Arrows point to positive staining in the stroma.
